# Lactylation: From Homeostasis to Pathological Implications and Therapeutic Strategies

**DOI:** 10.1002/mco2.70226

**Published:** 2025-05-29

**Authors:** Xi Chen, Yixiao Yuan, Fan Zhou, Lihua Li, Jun Pu, Yong Zeng, Xiulin Jiang

**Affiliations:** ^1^ Key Laboratory of Neurological and Psychiatric Disease Research of Yunnan Province The Second Affiliated Hospital of Kunming Medical University Kunming China; ^2^ NHC Key Laboratory of Drug Addiction Medicine Kunming Medical University Kunming China; ^3^ Department of Medicine, UF Health Cancer Center University of Florida Gainesville Florida USA; ^4^ Department of Hematology the Second Hospital Affiliated to Kunming Medical University Kunming China

**Keywords:** biological significance, cancer immunotherapy, pathology, physiology, protein lactylation

## Abstract

Lactylation, a recently identified post‐translational modification, represents a groundbreaking addition to the epigenetic landscape, revealing its pivotal role in gene regulation and metabolic adaptation. Unlike traditional modifications, lactylation directly links metabolic intermediates, such as lactate, to protein function and cellular behavior. Emerging evidence highlights the critical involvement of lactylation in diverse biological processes, including immune response modulation, cellular differentiation, and tumor progression. However, its regulatory mechanisms, biological implications, and disease associations remain poorly understood. This review systematically explores the enzymatic and nonenzymatic mechanisms underlying protein lactylation, shedding light on the interplay between cellular metabolism and epigenetic control. We comprehensively analyze its biological functions in normal physiology, such as immune homeostasis and tissue repair, and its dysregulation in pathological contexts, including cancer, inflammation, and metabolic disorders. Moreover, we discuss advanced detection technologies and potential therapeutic interventions targeting lactylation pathways. By integrating these insights, this review aims to bridge critical knowledge gaps and propose future directions for research. Highlighting lactylation's multifaceted roles in health and disease, this review provides a timely resource for understanding its clinical implications, particularly as a novel target for precision medicine in metabolic and oncological therapies.

## Graphical abstract

Lactylation, a novel post‐translational modification derived from lactate metabolism, plays a critical role in cellular homeostasis and disease pathogenesis. Under physiological conditions, lactylation regulates gene expression, immune responses, and metabolic adaptation. However, dysregulated lactylation is implicated in various pathological processes, including cancer progression, inflammatory diseases, and metabolic disorders. Targeting lactylation pathways presents potential therapeutic opportunities, with emerging strategies focusing on metabolic intervention, enzymatic regulation, and epigenetic modulation. This review provides a comprehensive overview of lactylation's biological significance, its implications in disease, and the latest advances in therapeutic targeting.

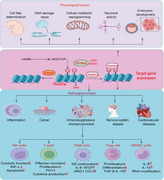

## 1 Introduction

Post‐translational modifications (PTMs) are crucial regulators of protein function, mediating diverse cellular processes and enabling rapid adaptation to physiological and environmental changes [1]. Among the myriad of PTMs, lysine lactylation, first identified in 2019, has emerged as a novel epigenetic mechanism that directly links cellular metabolism to gene regulation [2]. Unlike classical PTMs, which rely primarily on enzymatic modification, lactylation is unique in utilizing lactate, a byproduct of glycolysis, as a substrate, thereby bridging metabolic flux and epigenetic control. This discovery has sparked significant interest in understanding its regulatory mechanisms and implications in health and disease. Recently, scholars have proposed the concept of the “lactate clock,” which suggests that when endogenous or exogenous lactate reaches a critical threshold concentration within the cell, Kla is triggered [3]. This concept provides insights into the mechanisms controlling the initiation and cessation of Kla [4]. Acylation modifications, including Kla, regulate protein activity and conformation by covalently attaching chemical groups or small molecules to amino acid side chains [5, 6]. Specifically, for Kla, the lactyl group covalently binds to lysine residues through enzymatic or nonenzymatic pathways [2, 7].

Recent studies have revealed that lactylation is implicated in various biological functions, including macrophage polarization [8], chromatin remodeling [9], and cellular differentiation [10]. Its dysregulation has been associated with pathological conditions such as cancer progression [11], inflammatory responses [12], and metabolic disorders [13]. Despite these advances, the molecular machinery underlying lactylation and its comprehensive biological roles remain largely unexplored. Furthermore, the lack of robust detection methods and specific inhibitors targeting lactylation pathways poses challenges for in‐depth investigations and therapeutic development.

This review aims to provide a comprehensive overview of the current knowledge on protein lactylation, highlighting its mechanisms, biological functions, and potential clinical applications. By synthesizing the latest findings, we discuss its enzymatic and nonenzymatic regulatory mechanisms, the interplay between lactylation and other PTMs, and its roles in metabolic adaptation and disease pathogenesis. Additionally, we explore innovative detection technologies and emerging therapeutic strategies that target lactylation pathways.

The structure of this review is designed to guide readers through the multifaceted aspects of lactylation. We begin with an overview of the discovery and biochemical basis of lactylation, followed by an in‐depth examination of its biological functions in health and its aberrant roles in disease. Subsequently, we address technological advancements in detecting lactylation and potential therapeutic interventions. By integrating these insights, this review aims to bridge critical knowledge gaps and inspire future research into the mechanisms and applications of this intriguing PTM.

## 2 PTMs of Proteins

**FIGURE 1:**
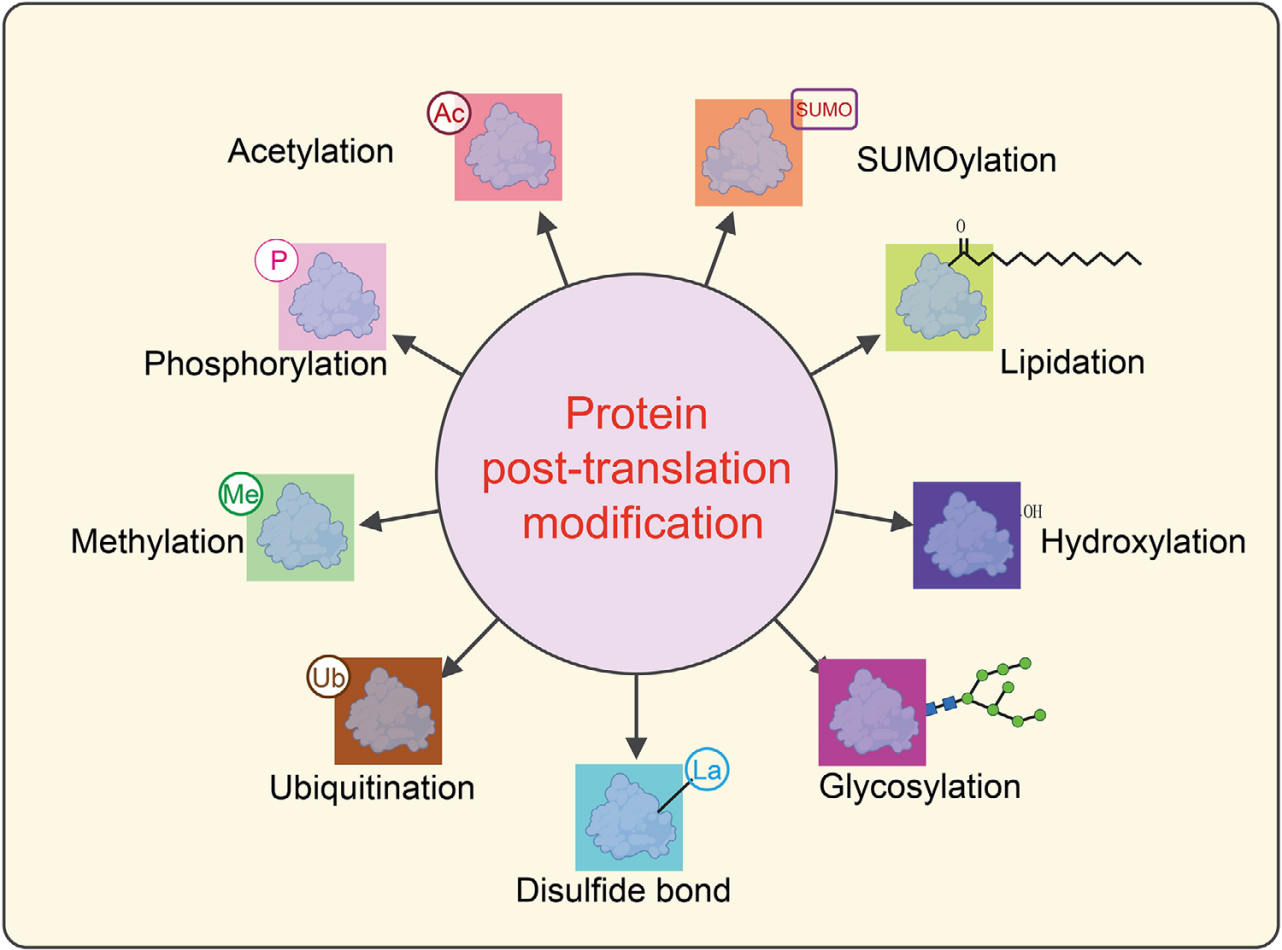
Classification of post‐translational modifications of proteins. This schematic diagram illustrates various types of post‐translational modifications (PTMs) that proteins undergo, which significantly impact their structure, function, and interactions. At the center, “Protein post‐translation modification” is highlighted in a pink circle, with arrows pointing outward to different types of PTMs. Each modification is represented by a colored box containing a protein structure with an associated chemical tag: Acetylation (Ac): The addition of an acetyl group (‐COCH₃) to lysine residues, often regulating gene expression by modifying histones. Represented in a red box. SUMOylation (SUMO): The attachment of Small Ubiquitin‐like Modifier (SUMO) proteins, affecting nuclear transport, transcriptional regulation, and protein stability. Shown in an orange box. Lipidation: The addition of lipid moieties, such as fatty acids or prenyl groups, which facilitate membrane association and protein localization. Represented in a green box with a lipid tail symbol. Hydroxylation (‐OH): The enzymatic addition of hydroxyl groups, often occurring on proline or lysine residues, which is crucial for collagen stability and hypoxia signaling. Shown in a dark blue box. Glycosylation: The attachment of carbohydrate groups, influencing protein folding, stability, and cell−cell communication. Represented in a magenta box with a glycan structure. Disulfide bond (La): The formation of covalent bonds between cysteine residues, stabilizing protein tertiary and quaternary structures. Displayed in a cyan box. Ubiquitination (Ub): The covalent attachment of ubiquitin molecules, marking proteins for degradation via the proteasome or regulating cellular signaling. Shown in a brown box. Methylation (Me): The addition of methyl groups to lysine or arginine residues, influencing chromatin structure and transcriptional activity. Represented in a green box. Phosphorylation (P): The addition of phosphate groups to serine, threonine, or tyrosine residues, playing a key role in signal transduction and cellular regulation. Displayed in a light yellow box.

When cellular metabolism undergoes reprogramming, shifting from oxidative phosphorylation to glycolysis, the structure and function of intracellular proteins adapt accordingly [14]. Through PTMs, proteins alter their physicochemical properties by adding chemical groups to amino acid residues [15]. This induces changes in spatial structure, increases structural complexity, and subsequently regulates activity, thereby enriching the diversity and functionality of proteins [16]. Common types of PTMs include acetylation, methylation, SUMOylation, lipidation, hydroxylation, methylation, glycosylaton, phosphorylation, ubiquitination, and acylation all of which play crucial roles in various physiological and pathological processes (Figure 1) [17]. Given that the primary focus of this article is protein lactylation, we have only briefly summarized the common PTMs of proteins, along with their regulatory enzymes and biological functions (Figure 1).

Among these various protein modifications, acylation is particularly widespread. Protein acylation is a significant PTM, wherein different types of acyl groups are covalently attached to specific sites on proteins, such as lysine or cysteine residues, to regulate their structure and function [18, 19, 20]. These modifications mainly include acetylation [21], propionylation [22], butyrylation [23], succinylation [24], crotonylation [25], and lactylation [26]. These different acylation modifications are extensively involved in biological processes such as cell signaling, metabolic regulation, gene expression, and disease development [12].

Lactylation, in particular, forms a complex regulatory network in both time and space with other acylation modifications, such as acetylation, propionylation, succinylation, and phosphorylation [27]. These modifications often target the same amino acid residues and affect protein activity, stability, and localization, suggesting functional similarities and potential crosstalk among them [28]. This indicates that Kla not only functions independently but can also interact in complex ways with other PTMs. Such interactions coordinate cellular processes and highlight the integrative role of Kla in regulating cellular functions. For example, acetylation and lactylation: both modifications occur on lysine residues and may competitively occupy the same modification sites, thus influencing the levels and functions of lactylation [29]. Methylation and lactylation: methylation, which modifies the side‐chain amino group of lysine, may prevent the attachment of the lactyl group, indirectly interfering with lactylation [30]. Ubiquitination and lactylation: ubiquitination may regulate the stability of target proteins, thereby indirectly altering the expression or function of lactylated proteins [31]. These interferences may not only alter the distribution and levels of lactylation but could also impact the cell's metabolic state or signaling pathways through downstream effects, potentially exacerbating the onset and progression of certain diseases.

## 3 Introduction to Protein Lactylation Modifications

In 2019, Zhang et al. discovered that histones in human and mouse cells could undergo lactylation modifications, revealing that histone lactylation could influence the regulation of gene expression in chromatin [2]. This discovery initiated a new chapter in lactylation modification research and highlighted a novel direction for studying the metabolism‐epigenetics axis.

### 3.1 Discovery of Protein Lactylation Modifications

In late 2019 and early 2020, two research groups independently discovered protein lactylation (lactylation or lactylation, Kla) as a novel PTM [2]. Zhang et al. first employed mass spectrometry to detect a 72.021 Da mass shift on lysine residues of histones. Using isotope metabolic labeling techniques and various in vivo and in vitro experiments, they confirmed the widespread existence of histone lysine lactylation modifications. Subsequently, Gaffney et al. also demonstrated protein lactylation through mass spectrometry [7]. Notably, these two independent groups held different views regarding the properties of lactylation, substrate sources, and the target proteins subjected to this modification. First, Zhang et al. posited that lactylation is an active enzymatic PTM process using lactyl‐CoA as the substrate [2]. Recent studies have detected the presence of lactyl‐CoA in mammalian cells and tissues via liquid chromatography‐tandem mass spectrometry [32]. In contrast, Gaffney et al. suggested that lactylation is a passive nonenzymatic acyl transfer process using lactoyl‐glutathione (LGSH) as the substrate, with the cellular level of LGSH being regulated by glyoxalase II (GLO2) [9]. Second, the former focused primarily on histone lactylation, elucidating its role as a novel epigenetic regulator of gene transcription, while the latter found that many metabolic enzymes were also lactylated, with this modification providing negative feedback regulation of the glycolytic pathway [33]. Subsequent studies confirmed that lactylation occurs on both histones and many nonhistone proteins. Although some unresolved discrepancies remain, the discovery of protein lactylation has not only opened a new field in PTM research but also proposed potential molecular mechanisms for the role of lactate in physiological and pathological processes such as tumor biology, metabolism, and immunity [34].

L‐lactylation was initially considered a PTM associated with histone modifications. However, with the progression of research, scientists have discovered that L‐lactylation is not only present on histones but is also widely found in nonhistone proteins, suggesting that its role within the cell may extend beyond epigenetic regulation [35]. Nonhistone lysine lactylation (L‐lactylation) is an emerging PTM, primarily occurring on lysine residues through the acylation of lactate. Nonhistone lysine lactylation has been identified in hepatocellular carcinoma (HCC), where it promotes tumor proliferation and metastasis [36]. This highlights the critical role of lysine lactylation as a key link between lactate metabolism, tumor metabolism, and patient prognosis. Previous studies have confirmed that the nonhistone lactylation of the protein METTL16 enhances therapeutic efficacy by promoting cuproptosis [37]. Recent studies have also revealed several nonhistone Kla sites in non‐small cell lung cancer (NSCLC), which are associated with tumor metastasis and resistance to immunotherapy. Further research has shown that lactate induces lactate‐APOC2‐K70, triggering the release of free fatty acids into the extracellular space and promoting the accumulation of Treg cells [38]. This interaction contributes to immune therapy resistance and tumor metastasis. These findings underscore the role of nonhistone lysine lactylation in tumor progression and suggest its potential as a biomarker for predicting resistance to immunotherapy.

### 3.2 Writers and Erasers of Protein Lactylation Modifications

As a widely occurring and evolutionarily conserved type of PTM, lysine acylation modifications are often dynamic in a spatiotemporal manner [39]. Galligan et al. proposed that LGSH serves as an acyl donor for protein lactylation independently of any enzymes, as lysine lactylation was detectable after coincubation of histone H4 with LGSH [7]. Contrary to Gaffney et al., many lysine acylation modifications require specific proteins to “write” and “erase” them. Histone acetyltransferases (HATs) and histone deacetylases (HDACs) are involved in the “writing” and “erasing” of various lysine acylation modifications (such as acetylation and succinylation) [40]. Studies have shown that HATs and HDACs also participate in the dynamic regulation of protein lactylation. Overexpression of HAT p300 in human embryonic kidney 293T (HEK293T) cells significantly enhanced the global lactylation levels of histones, whereas knockout of the EP300 gene, which encodes p300, reduced global histone lactylation and H3K18la levels in human colon cancer HCT116 cells and HEK293T cells [41]. Similarly, the knockdown of p300 in murine bone marrow‐derived macrophages significantly weakened lactate‐induced histone lactylation. Another study showed that lactate could induce increased lactylation of high mobility group protein B1 (HMGB1) in murine macrophage cell line RAW 264.7, a phenomenon significantly antagonized by the p300/CREB‐binding protein (CBP) inhibitor C646, and also markedly inhibited by p300 or CBP knockdown [29]. In vitro chromatin histone modification and transcription assays also demonstrated that tumor protein p53 (TP53) and p300 jointly promote the lactylation of histones H3 and H4. While the indirect role of p300 and CBP in protein lactylation within cells cannot be entirely ruled out, these in vivo and in vitro experiments suggest that p300 and CBP may serve as “writer” proteins for lactylation, potentially functioning individually or cooperatively to accomplish some protein lactylation modifications. Additionally, Zhao et al. reported for the first time that Class I histone deacetylases (HDAC1‐3) and Class III histone deacetylases (SIRT1‐3) are the most effective lysine lactylation “erasers” in vitro, with overexpression and knockdown experiments indicating that HDAC1 and HDAC3 exert delactylation functions within cells [42]. A previous interesting study confirmed that HBO1 is a multifunctional histone acyltransferase, which not only catalyzes histone acetylation but also catalyzes propionylation, butyrylation, and crotonylation both in vivo and in vitro [43]. Similarly, a landmark study in April 2024 discovered that alanyl‐tRNA synthetase (AARS1), acting as a lactate receptor and lactylase, can bind to lactate and catalyze the formation of lactyl‐AMP, subsequently transferring the lactyl group to lysine residues, thereby inducing protein lactylation. This modification affects the function of various proteins, including the tumor suppressor p53. AARS1 primarily targets p53, acetylating its lysine residues at positions K120 and K139, thereby inhibiting its DNA‐binding and transcriptional activation abilities, ultimately reducing its tumor suppressor functions [44]. Moreover, the study proposed a potential therapeutic strategy whereby β‐alanine interferes with the binding of AARS1 to lactate, reducing p53 acetylation and mitigating tumor development in animal models. Additionally, the research revealed that beyond its classical function as an alanyl‐tRNA synthetase, AARS1 can sense intracellular lactate levels and act as a lactyl transferase, using lactate directly as a lactyl donor to catalyze the lactylation of proteins such as YAP‐TEAD. This nonclassical function of AARS1 links the high levels of lactate in tumor cells to the YAP‐driven malignant proliferation signaling, offering a new perspective for understanding the Warburg effect in tumor cells [45]. Recent studies have shown that nuclear GTPSCS can function as a lactoyl‐CoA synthetase, with GTPSCS/p300 coregulating histone H3K18la and GDF15 expression, thereby promoting glioma proliferation and radioresistance [46].

### 3.3 Identification of Protein Lactylation Sites

Histones are fundamental components of chromatin, with core histones (H2A, H2B, H3, and H4) and the linker histone H1 playing essential roles in DNA packaging and gene regulation [47]. These proteins are rich in lysine residues, which serve as hotspots for diverse PTMs such as acetylation, methylation, ubiquitination, crotonylation, and lactylation. The versatility of lysine residues in accommodating multiple PTMs underscores their critical role in regulating chromatin dynamics and gene expression [48]. Histone PTMs contribute to the “histone code,” a complex regulatory system that influences various cellular processes, including transcription, replication, and DNA repair [49]. Notably, histone lactylation has emerged as a novel PTM linked to metabolic states, particularly under hypoxic and glycolytic conditions. The addition of a brief overview of histones, their PTM‐rich lysine residues, and the interplay of various PTMs would provide a more holistic context for understanding the significance of lactylation in epigenetic regulation and its broader implications in cellular physiology. Zhang et al. first identified 26 and 18 lysine lactylation sites on core histones in human cervical cancer HeLa cells and murine bone marrow‐derived macrophages, respectively. Subsequent studies identified 16, 16, 6, and 14 lysine lactylation sites on histones in mouse brain tissues, Trypanosoma brucei, Botrytis cinerea, and rice, respectively [2]. The differences in identified histone lactylation sites among these studies might stem from species and tissue specificity or the spatiotemporal dynamics of lactylation. The chromosomal regions enriched with these histone lactylation sites and their associated biological processes remain unclear.

Zhang et al. provided the first comprehensive description of lactylation modifications. They found that during inflammation, M1 phenotype macrophages are rapidly activated, producing large amounts of proinflammatory cytokines and inducing genes such as NOS2, accompanied by the Warburg effect. Over time, however, macrophages need to polarize to the M2 phenotype to help repair collateral damage caused by inflammation [50]. At this stage, lactylation modifications occur on core histones and accumulate in the promoters of homeostatic genes, directly inducing the transcription of chromatin genes, such as arginase 1 (Arg1), which is involved in wound healing. This facilitates the transition of macrophages from the M1 to M2 phenotype [51]. In addition, studies on mice subjected to social defeat stress (SDS) identified 63 lactylated proteins in the prefrontal cortex, including five subtypes of linker histone H1 (H1.1–1.5). Pan‐lactylation modifications were detected in glutamatergic neurons, GABAergic neurons, astrocytes, and microglia [39]. Similar findings were observed in mouse oocytes and preimplantation embryos, where pan‐lactylation of histones, as well as H3K23la and H3K18la, were abundant, peaking at the blastocyst stage [52]. Furthermore, lactylation levels were significantly elevated in clinical brain samples from Alzheimer's disease (AD) patients and mouse brain tissues, with H4K12la being notably prominent, suggesting a potential target for AD treatment [53]. Studies on Toxoplasma gondii revealed lactylation modifications in histone variants such as H2A1 and H2AX [54].

**FIGURE 2:**
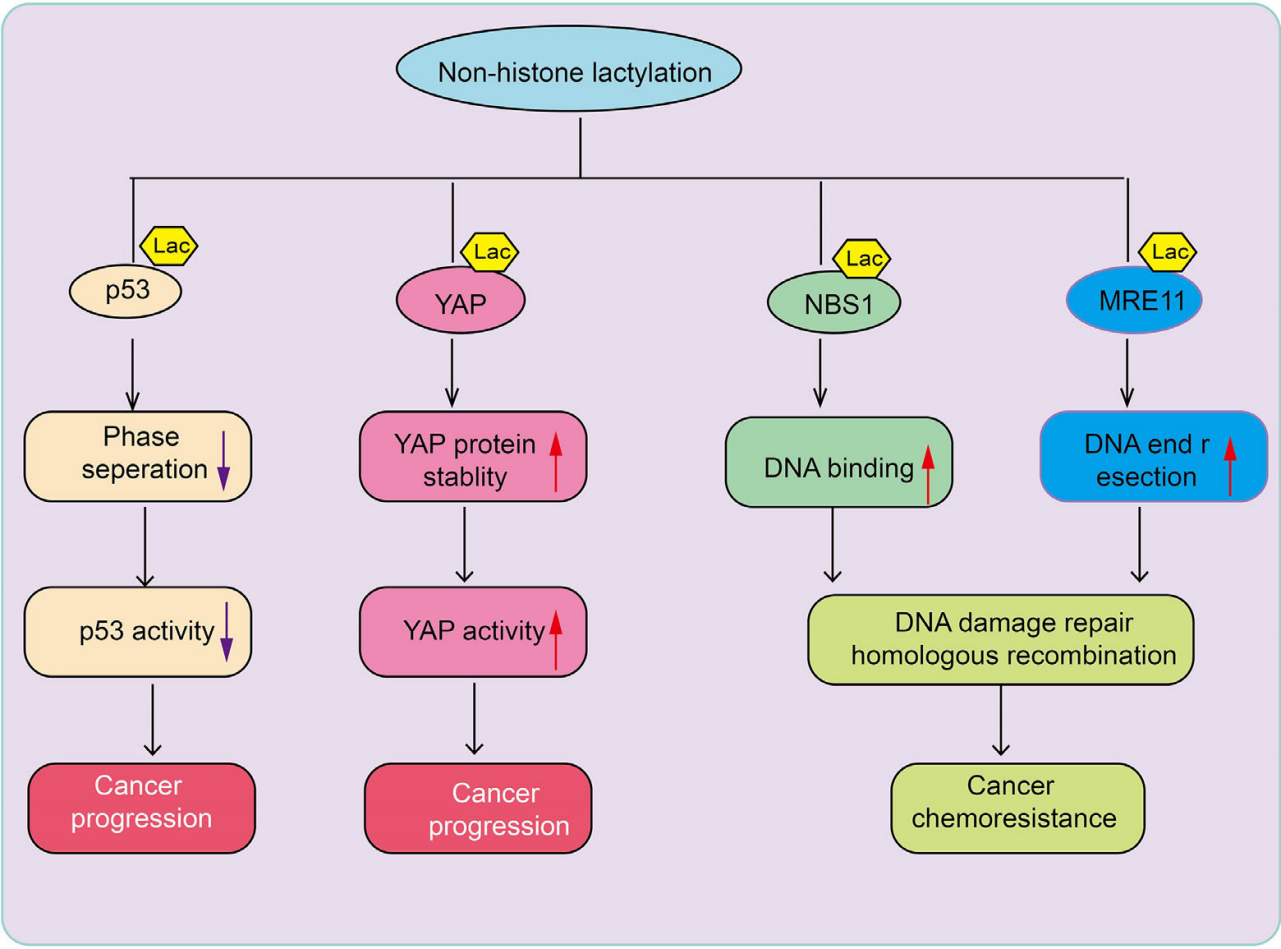
The role of nonhistone lactylation in cancer progression and chemoresistance. This schematic diagram illustrates the effects of nonhistone lactylation on key cancer‐related proteins, including p53, YAP, NBS1, and MRE11, highlighting its role in cancer progression and chemoresistance. The lactylation of p53 reduces phase separation, leading to decreased p53 activity. As a tumor suppressor, reduced p53 activity contributes to cancer progression. n: Lactylation of YAP enhances its protein stability, leading to increased YAP activity. YAP is a key effector in the Hippo signaling pathway, and its activation promotes cancer progression. The lactylation of NBS1 enhances its DNA‐binding ability, facilitating homologous recombination‐mediated DNA damage repair. This process contributes to cancer cell survival and chemoresistance. The lactylation of MRE11 enhances DNA end resection, promoting DNA damage repair through homologous recombination. This mechanism supports cancer cell survival under therapeutic stress, leading to chemoresistance.

Lactylation modifications can occur not only on histones but also on nonhistone proteins, although lysine residues on nonhistone proteins are less prone to lactylation. Research indicates that in the fungus Botrytis cinerea, lactylated proteins are predominantly distributed in the nucleus (36%), mitochondria (27%), and cytoplasm (25%), participating in various cellular processes [55]. Among these, 43 ribosomal proteins undergo lactylation, playing crucial roles in protein translation regulation and ribosome assembly. Numerous lactylated proteins have been identified in protozoan parasites, engaging in trans‐splicing, cap binding, and RNA export, translation, and degradation. Key enzymes involved in glycolysis, such as aldolase (ALDO), phosphoglycerate kinase (PGK), and pyruvate kinase (PYK), are also subject to lactylation [56, 57]. In septic mouse models, lactylated high mobility group box protein 1 (HMGB1) is released from macrophages via exosomes, increasing endothelial permeability and decreasing survival rates in septic mice [29]. Hyperlactylation of poly(ADP‐ribose) polymerase 1 (PARP1) regulates its ADP‐ribosylation activity, potentially aiding DNA repair. Lactylation can also occur directly on the CCCH‐type zinc finger domain of human METTL3 protein, mediating tumor immune evasion [58]. Lactylation modifications mainly occur on proteins with a molecular weight of 25 kDa, and lactylation sites tend to be located in α‐helix structures rather than β‐helix or disordered regions. These findings suggest that lactylation is widespread among various proteins, particularly enriched in energy metabolism pathways, indicating it may be a fundamental modification process within organisms [59]. Lactylation of nonhistone proteins is believed to enhance or inhibit their functions. For instance, AARS1 senses lactate and translocates to the nucleus to lactylate YAP, maintaining its nuclear localization and promoting YAP activation. AARS1‐mediated lactylation of p53 suppresses its phase separation, DNA binding, and transcriptional activation, thereby promoting tumorigenesis [45] (Figure 2). Lactylation is also associated with DNA repair and chemotherapy resistance in cancer. Key components of the MRE11‐RAD50‐NBS1 (MRN) complex, which is responsible for maintaining genome stability, such as MRE11 and NBS1, are lactylated. This modification is crucial for the formation of the complex and subsequent DNA repair [60] (Figure 2).

Liquid chromatography‐mass spectrometry is an important technique for elucidating the lactylation modification landscape. Using this technology, Gaffney et al. identified 350 lactylated proteins in HEK293T cells, primarily concentrated in glycolysis and carbon metabolism pathways [36]. A Japanese research group identified 63 lactylated proteins in mouse brains (specific sites not detailed), with lactylation of 12 proteins altered during SDS in mice [39]. Chinese researchers identified 273 lysine lactylation sites in 166 proteins in Botrytis cinerea [61]. Notably, 43 ribosomal structural proteins were found to be lactylated, suggesting the involvement of lactylation in protein translation [61]. Zhang et al. identified 387 lactylation sites in 257 proteins in Trypanosoma brucei, with 14 lysine residues on heat shock protein 90 undergoing lactylation [62]. Additionally, another study identified 638 lysine lactylation sites in 342 proteins in developing rice grains [63]. Beyond these studies, lactylation and its regulation have also been observed in individual proteins. Xiong et al. identified lysine lactylation at positions 281 and 345 on methyltransferase‐like 3 (METTL3) in HEK293T cells, enhancing its binding ability to N6‐methyladenosine (m6A)‐modified RNA [64]. Although specific lactylation sites were not identified in murine macrophage cell line RAW 264.7, researchers found that exogenous lactate could significantly elevate HMGB1 lactylation [29]. These studies collectively reveal that besides histones, numerous proteins in the nucleus, cytoplasm, mitochondria, endoplasmic reticulum, and cell membrane undergo lactylation, suggesting that protein lactylation might regulate various life activities. Compared to time‐consuming experimental methods, computational prediction of protein lactylation sites is more convenient and efficient. Chinese researchers have designed a predictor named “FSL‐Kla” (http://kla.zbiolab.cn/), which aids researchers in predicting potential lactylation sites on proteins of interest [65].

### 3.4 Pathways of Lactylation

Lactate exists in two stereoisomers, L‐lactate and D‐lactate. Correspondingly, lactylation also follows two pathways: L‐lactylation and D‐lactylation, also referred to as direct and indirect lactylation [35, 66]. Given the widespread research and significance of L‐lactylation, this review focuses on the recent advances in the study of L‐lactylation. L‐lactylation is a novel PTM driven by the metabolic product L‐lactate. This modification primarily occurs on lysine residues and is closely linked to cellular metabolic states [67] Lysine L‐lactylation (Kl‐la) includes three isomers: KL‐la, N‐ε‐(carboxyethyl)‐lysine (Kce), and D‐lactyl‐lysine (Kd‐la). Studies have shown that KL‐la, dynamically regulated by glycolysis, is the predominant lactylation isomer on histones, whereas Kd‐la and Kce appear only under conditions where the glycolytic pathway is incomplete. Moreover, lactoyl‐CoA has been identified as a precursor for L‐lactylation, and its levels are positively correlated with the expression of KL‐la. The discovery of L‐lactylation provides an important tool for exploring the connection between metabolism and epigenetic regulation and may offer new therapeutic targets for metabolic‐related diseases, such as cancer [35].

### 3.5 Temporal and Reversible Nature of Lactylation Modifications

Lactylation modifications exhibit temporal characteristics. Histone lactylation increases in a time‐dependent manner during M1 macrophage polarization (within 24 h). Following M1 macrophage stimulation, histone lactylation at gene promoters increases with a delay, resulting in the activation or reactivation of specific H3K18la genes between 16 and 24 h, inducing the transition of M1 macrophages to an M2 phenotype [68]. This delayed temporal dynamic effect has been metaphorically described as a “lactate clock.” During the promotion of pluripotency reprogramming, there is a strong temporal correlation between increased expression of the transcription factor Glisl and changes in lactylated histones [64]. However, some researchers suggest that these data require independent validation. Lactylation modifications are dynamic and reversible processes, mediated by deacetylases. Under the combined regulation of acetyltransferases and deacetylases, lactylation modifications present a dynamic and reversible process.

## 4 Factors Influencing Lactylation

The level of lactylation can be influenced by various factors, including the production and transport of substrate lactate, the acyltransferases providing the lactyl group, neural excitation, gene expression, and the stimulation of certain traditional Chinese medicine components (Table 1).

**TABLE 1:** Summary of regulatory factors of lactylation.

Regulatory factor	Function	Ref
Lactate	Rotenone to promote glycolysis can increase intracellular lactate levels and histone lactylation	[26]
Lactate transport	MCTs facilitate macrophage uptake of extracellular lactate for HMGB1 lactoylation	[29]
Acetyltransferases	The p300 enzyme functions as a “writer” enzyme in the enzymatic process of histone lactoylation	[69]
Deacetylases	Histone deacetylases can act as “eraser” enzymes to delactoylate	[70]
Transcription factor	Transcription factor Glis1 can enhance histone lactoylation (specifically H3K18la) expression	[71]
Neural excitation	Neural excitation can elevate histone H1 lactylation levels	[72]
Demethylzeylasteral	Demethylzeylasteral inhibits histone modification sites, H3K9la and H3K56la	[73]

### 4.1 Increased Lactate Production and Lactylation

Several studies have demonstrated that lysine lactylation levels can increase in a dose‐dependent manner with rising intracellular lactate concentrations [74]. Zhang et al. proposed that lactylation is derived from lactate; methods such as using gamma‐interferon combined with lipopolysaccharide or bacterial stimulation can increase lactate production in cells [2]. Additionally, using rotenone, a mitochondrial respiration inhibitor, to promote glycolysis can increase intracellular lactate levels and histone lactylation. Conversely, inhibiting glycolysis with the nonmetabolizable glucose analog 2‐deoxy‐D‐glucose (2‐DG) can prevent lactate production, thereby reducing histone lactylation levels [75]. The study also indicated that exogenous sodium lactate could promote histone lactylation, but the lactylation still depends on the cell's ability to produce endogenous lactate. Lactate concentration is also related to oxygen levels, with hypoxic conditions leading to increased lactate production. Yang et al. compared lactylation under different oxygen levels in embryos and found that histone lactylation is influenced by the oxygen concentration in the culture environment, showing a significant reduction under hypoxic conditions (2% O2) [11]. Hypoxic in vitro cultures can reduce histone lactylation, though some scholars noted the difficulty in detecting results due to reduced cellular activity under hypoxic conditions, and others suggested that hypoxic in vitro cultures might interfere with normal gene expression and affect experimental outcomes [76]. Additionally, studies on high‐altitude adaptation in mice have shown increased lactylation levels in eye proteins with the number of days of high‐altitude exposure, possibly due to increased lactate production in low‐oxygen environments at high altitudes. Lactate production is a key step in glycolysis, and lactate levels influence both glycolysis and lactylation. There is a positive correlation between lysine lactylation levels and glycolysis rate [77]. Pan et al. found that a positive feedback loop of “glycolysis‐lactate‐histone lactylation‐glycolysis” promotes AD progression in patients and mice microglia. Lactate dehydrogenase (LDHA), a redox enzyme in glycolysis that catalyzes the oxidation of lactate to pyruvate, has been shown to decrease lactylation levels when its activity is inhibited [53]. Yang et al. detailed that inhibiting LDHA A expression significantly reduces histone lactylation in human renal clear cell carcinoma and renal cancer cells, while silencing the LDHA B gene has a minor effect, indicating different phenotypes of LDHA affect lactylation differently [78]. In vitro simulation experiments also showed that bone marrow‐derived macrophages lacking B cell adaptors for phosphatidylinositol 3‐kinase (BCAP) cannot effectively use lactate for histone lactylation, which is necessary for the expression of repair genes [78]. This may be due to reduced expression of key glycolytic enzymes such as hexokinase and LDHA A in the absence of BCAP, leading to decreased lactate production, although adding exogenous sodium lactate can mitigate this [79].

### 4.2 Lactate Transport and Lactylation

Both L‐lactate and D‐lactate can be transported in and out of cells by the monocarboxylate transporter (MCT) family [80]. L‐lactate transport is primarily mediated by MCT1, MCT2, and MCT4, with MCT4 promoting lactate efflux and MCT1 and MCT2 promoting lactate influx. The direction of transport is determined mainly by the lactate concentration gradient [81]. Yang et al. confirmed that MCTs facilitate macrophage uptake of extracellular lactate for HMGB1 lactylation, and using the MCT inhibitor CHC to block cellular uptake of extracellular lactate inhibited the high levels of lactylation induced by elevated lactate [29]. However, Hagihara et al. induced histone H1 lactylation in mouse neuronal cells using different methods and then treated them with the MCT2 inhibitor α‐cyano‐4‐hydroxycinnamate (4‐CIN) and the more selective MCT1/2 inhibitor AR‐C155858, finding no significant changes in lactylation levels, suggesting the possible concurrent occurrence of an indirect lactylation pathway not inhibited by MCT inhibitors [82].

### 4.3 Regulation of Lactylation by Acetyltransferases and Deacetylases

Histone lactylation is catalyzed by regulatory enzymes rather than occurring as a spontaneous chemical reaction [83]. The rate of lactylation largely depends on the concentration of lactyl‐CoA, which provides the lactyl group, and the concentration of HATs, which catalyze the transfer of the lactyl group, though the specific concentrations require further investigation [84]. In microorganisms, CoA synthetase or transferase can catalyze lactate to form lactyl‐CoA, and lactyl‐CoA is present in small amounts in animal cells, but its specific formation mechanism remains unclear [85]. The acetyltransferase family has broad acyltransferase activity, utilizing different acyl‐CoAs as substrates for lysine acylation of various histones. The p300 enzyme is a multisubstrate acetyltransferase, and multiple experiments have confirmed its role as a “writer” enzyme in the enzymatic process of histone lactylation. Additionally, the p300 homolog CREB‐binding protein (CBP) also has this enzymatic function [86]. In contrast to promoting lactylation, HDACs can act as “eraser” enzymes to delactoylate. There are 18 types of HDACs, divided into two classes: Zn2+‐dependent deacetylases, including class I, II, and IV enzymes composed of HDAC1‐11, with HDAC1‐3 showing strong de‐L‐lactylation and de‐D‐lactylation activity; and NAD+‐dependent deacetylases, including class III sirtuin proteins 1–7, with SIRT1‐3 exhibiting strong de‐L‐lactylation activity [87]. However, HDAC1‐3 has a thousand‐fold higher enzymatic efficiency than SIRT2, making them the primary deacetylation enzymes [88].

### 4.4 Gene Expression and Lactylation

Lactylation modification not only regulates gene expression but is also influenced by it, creating a feedback loop within the organism. For instance, the transcription factor Glis1 in the early stages of reprogramming can shut down somatic gene expression, initiate glycolytic gene expression, promote metabolic remodeling, increase lactate and acetyl‐CoA production, and enhance histone lactylation (specifically H3K18la) expression, constituting a “cross‐border cascading reaction” of “epigenome‐metabolome‐epigenome” [89]. The inactivated VHL tumor suppressor gene can also trigger a positive feedback loop of “histone lactylation‐PDGFRβ‐histone lactylation,” promoting the progression of clear cell renal cell carcinoma (ccRCC), with high levels of histone lactylation indicating poor prognosis for patients [90].

### 4.5 Increased Histone H1 Lactylation Due to Neural Excitation

Neural excitation induced by various causes can modulate lactylation levels, with higher levels of neuronal excitation correlating with increased lactylation. Experimental evidence has shown that mice subjected to SDS exhibit elevated levels of lactate and lactylation in the prefrontal cortex cells [39]. This mechanism is thought to involve neural excitation induced by SDS, which potentially increases lactate production and lactylation through intracellular metabolism and glycolytic pathways, preferentially elevating histone H1 lactylation levels. Moreover, neural excitation induced by high potassium ions or electroconvulsive shock also raises lactate and lactylation levels in mouse brain cells both in vivo and in vitro, suggesting that excessive neuronal activity underlies increased lactylation [39].

### 4.6 Demethylzeylasteral Reduces Histone H3 Lactylation

Demethylzeylasteral (DML), extracted from Tripterygium wilfordii, has been the first to demonstrate that components of traditional Chinese medicine can treat diseases through lactylation pathways [73]. Pan et al. reported that DML inhibits two tumorigenesis‐promoting histone modification sites, H3K9la and H3K56la, by suppressing histone H3 lactylation, thereby reducing the tumorigenicity of liver cancer stem cells [73]. Additionally, DML decreases intracellular lactate levels in a dose‐dependent manner, thus reducing histone lactylation in liver cancer stem cells [73].

### 4.7 Crosstalk Between Post‐Translational Modifications and Lactylation

Proteins undergo various PTMs, including phosphorylation, glycosylation, ubiquitination, methylation, acetylation, and lipidation, which influence one another. One PTM can promote or inhibit another, or work in combination, a phenomenon known as PTM crosstalk [91]. The study of lactylation's crosstalk with other modifications is still in its early stages. Evidence shows a close relationship between lactylation and acetylation. Histone lactylation and acetylation exhibit a high degree of spatial overlap, and lactylation levels increase as acetylation decreases. Additionally, lactylation and acetylation competitively bind to lysine residues on histone H3 in brain cells to regulate the expression of specific gene sets, with expression patterns determined by the balance between these two modifications [92]. Moreover, Yu et al. demonstrated the crosstalk between histone lactylation and m6A methylation in ocular melanoma for the first time. Lysine acylation's metabolic origins are also interconnected, highlighting a clear interplay between metabolism and epigenetics [93]. Lactylation serves as an intermediary linking metabolism and epigenetics, with crosstalk phenomena present in its modification process.

## 5 Physiological Functions of Protein Lactylation

**FIGURE 3:**
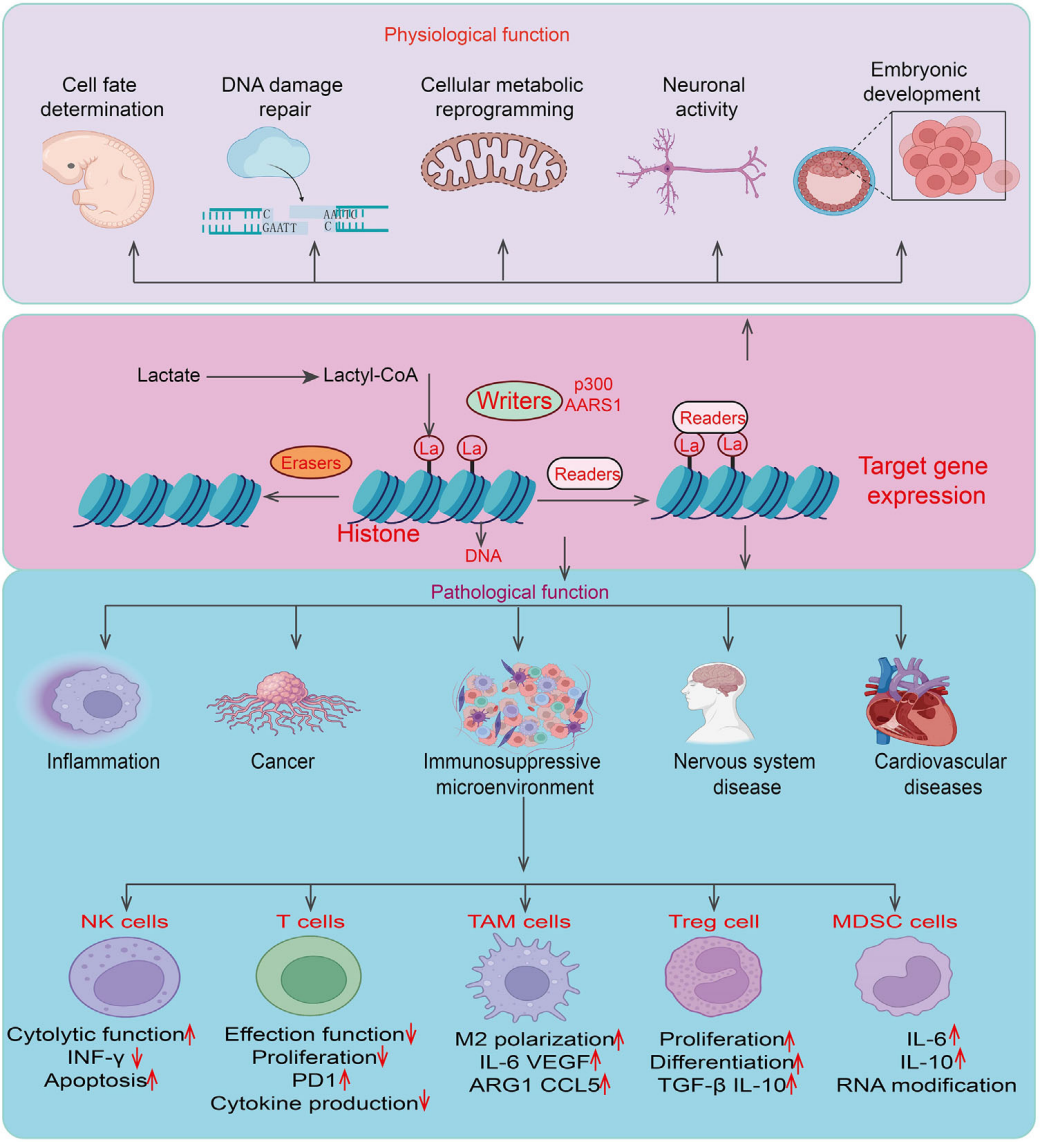
The physiological functions and pathological function of lactylation in process. Histone Lactylation Mechanism: Lactate is converted to lactyl‐CoA, which serves as a substrate for histone lactylation. Writers (e.g., p300, AARS1) catalyze the addition of lactyl groups to histones, while erasers remove these modifications. Readers recognize lactylated histones, leading to altered gene expression. Physiological Functions: Histone lactylation plays essential roles in cell fate determination, DNA damage repair, cellular metabolic reprogramming, neuronal activity, and embryonic development. These functions are crucial for normal cellular and organismal homeostasis. Pathological Functions: Aberrant histone lactylation is implicated in various diseases, including inflammation, cancer, immunosuppressive microenvironments, nervous system diseases, and cardiovascular disorders. Immune Regulation: Histone lactylation influences immune cell function: NK Cells: Decreased cytolytic function, reduced IFN‐γ production, and increased apoptosis. T Cells: Reduced effector function, decreased proliferation and cytokine production, and upregulated PD‐1 expression. Tumor‐Associated Macrophages (TAMs): Promotes M2 polarization, upregulating IL‐6, VEGF, ARG1, and CCL5, which contribute to an immunosuppressive microenvironment. Regulatory T Cells (Tregs): Enhances proliferation, differentiation, and secretion of immunosuppressive cytokines (TGF‐β, IL‐10). Myeloid‐Derived Suppressor Cells (MDSCs): Increases IL‐6 and IL‐10 expression and promotes RNA modification, further suppressing anti‐tumor immunity.

Protein lactylation refers to a PTM in which a lactyl group is added to lysine residues. This modification plays a crucial role in regulating cellular physiological functions through various mechanisms, making it an important type of PTM with broad biological significance. Here, we summarize the functions and mechanisms of protein lactylation in cell fate determination, embryonic development, lactylation and neuronal activity, and DNA damage repair (Figure 3).

### 5.1 Lactylation and Cell Fate Determination

Cell fate determination is a finely tuned and complex biological process involving multilevel regulation across the transcriptome, epigenome, and metabolome. During the reprogramming of mouse somatic cells, the maternal transcription factor Glis1 exerts two effects: repressing the expression of somatic genes and promoting the activation of glycolytic genes. Glis1‐induced metabolic reprogramming leads to increased lactate production and promotes histone lactylation (H3K18la) and pluripotency gene expression, thereby enhancing reprogramming efficiency and even achieving the reprogramming of senescent cells [76]. This research introduces a novel concept of an “epigenome‐metabolome‐epigenome” cross‐regulatory cascade in the regulation of pluripotent stem cell fate by Glis1 [76]. Furthermore, in vitro studies on mouse embryonic stem cells showed that lactate treatment could promote H3K18la modifications at promoters of lineage‐related and cleavage‐stage embryo‐related genes, leading to their widespread expression [76]. These studies highlight the critical role of histone lactylation in cell fate determination. Notably, during somatic cell reprogramming, H3K18la regulates the expression of pluripotency genes such as Oct4 and Mycn, while lactate treatment does not significantly alter the expression of pluripotency genes like Oct4 and Sox2 in mouse embryonic stem cells [94]. The reasons behind these differences warrant further investigation [94, 95].

### 5.2 Lactylation and Embryonic Development

From fertilization to preimplantation, histone PTMs undergo highly dynamic changes, and histone lactylation is a key component of this epigenetic reprogramming process [96]. Yang et al. reported that the levels of pan‐histone lactylation, H3K23la, and H3K18la are relatively low in zygotes and cleavage‐stage embryos after mouse fertilization but significantly increase in blastocyst‐stage embryos. This study provides the first description of the dynamic process of histone lactylation in mouse oocytes and preimplantation embryos [52]. They also found that low oxygen culture conditions reduce histone lactylation levels, impairing the developmental potential of preimplantation mouse embryos [52]. Beyond influencing embryonic development, lactylation also regulates endometrial receptivity to embryos. Researchers observed that H3K18la levels in the endometrium of pregnant sheep were significantly elevated, whereas they were markedly reduced in the endometrium of nonpregnant sheep. This suggests that glycolysis‐generated lactate in pregnant individuals promotes endometrial histone lactylation, epigenetically regulating the expression of redox homeostasis and apoptosis‐related genes, thereby enhancing endometrial receptivity and facilitating successful embryo implantation [11].

### 5.3 Lactylation and Neuronal Activity

Lactate plays various important roles in the brain physiological activities. Hagihara et al. discovered that neuronal excitation and SDS elevate lactate levels and protein lactylation in brain cells, identifying 12 proteins associated with SDS, including histone H1 [39]. The increased levels of protein lactylation correlate positively with the expression of the neuronal activity marker cFos and the reduction in social behavior and increase in anxiety‐like behaviors in mice under stress models [9]. Their study confirmed the presence of protein lactylation in the brain, suggesting that lactylation may play a critical role in regulating neuronal activity.

### 5.4 Lactylation and DNA Damage Repair

A groundbreaking study on July 3, 2024, revealed the crucial role of lactate in tumor resistance. Lactate enhances the DNA damage repair capability of tumor cells by promoting the lactylation of NBS1, enabling rapid repair after radiotherapy and chemotherapy damage, reducing treatment efficacy, and leading to resistance. This discovery identifies lactylation as a new target for combating tumor resistance. The study also found that stiripentol, a repurposed drug, inhibits lactate production, reducing NBS1 lactylation and disrupting the DNA repair mechanism in tumor cells, thereby enhancing the efficacy of radiotherapy and chemotherapy. Stiripentol shows high clinical translational value, offering a new therapeutic option for patients with treatment‐resistant tumors [69]. Another study found that low expression of LDHA is associated with downregulated homologous recombination repair, and further research identified MRE11 as having the highest lactylation levels. Lactylation at the MRE11 K673 site promotes MRE11's binding to DNA, facilitating DNA end resection and homologous recombination repair. Inhibition or mutation of MRE11 K673 lactylation significantly hinders the DNA repair process, with tumors exhibiting high MRE11 K673 lactylation being resistant to chemotherapy drugs. Inhibiting MRE11 K673 lactylation enhances the efficacy of chemotherapy, and a small molecule peptide specifically inhibiting MRE11 K673 lactylation significantly improves the tumor‐killing effect of platinum‐based or PARP inhibitor chemotherapy in vitro and PDX models. This research unveils the key molecular link between cellular lactate metabolism and homologous recombination repair, providing a new theoretical foundation and potential target for overcoming tumor resistance through DNA repair targeting [97].

## 6 Protein Lactylation Modifications and Pathological Processes

Protein lactylation plays a significant role in various diseases and pathological conditions, including inflammatory, tumor immune suppression microenvironment, neurodegenerative diseases, metabolic diseases, cardiovascular diseases, and diverse cancer progression. Understanding its mechanisms could provide insights into the pathogenesis of these diseases and potentially offer new avenues for diagnosis and treatment [8, 26, 27, 98, 99, 100] (Figure 3).

### 6.1 Lactylation and Inflammation

Lactate was once considered a metabolic waste product, but growing evidence suggests it plays a crucial regulatory role in immune cells. In 2019, Zhang et al. reported histone lysine lactylation, opening new research directions in how this novel PTM regulates the immune system, providing new insights into lactate's nonmetabolic functions and its involvement in infection and immunity [2]. Activated macrophages are generally classified into M1 macrophages, which are proinflammatory, and M2 macrophages, which are anti‐inflammatory. M1 macrophages typically have highly active glycolytic pathways. Therefore, it is conceivable that lactate levels significantly rise in bone marrow‐derived macrophages polarized to the M1 type after lipopolysaccharide and interferon‐γ stimulation. In bacterial infection models, histone lactylation (including pan‐lactylation and H3K18la) levels increase in the late stages of M1 macrophage polarization, inducing the expression of M2‐related genes such as arginase‐1 (Arg1). The authors proposed the existence of a “lactate clock” within macrophages, where lactate accumulation and enhanced histone lactylation in late‐stage M1 macrophages during bacterial infection trigger the expression of homeostatic genes, helping the host prevent additional tissue damage caused by inflammation [4]. The B cell adapter for phosphoinositide 3‐kinase (BCAP) also promotes the expression of genes involved in damage repair, such as Arg1, by regulating glycolysis and histone lactylation in macrophages. Similarly, lactate induces histone lactylation and Arg1 expression in alveolar macrophages. Moreover, elevated H3K18la levels in peripheral blood mononuclear cells of septic shock patients are significantly correlated with increased Arg1 transcription levels [101]. However, German researchers recently stated that although lipopolysaccharide induces lactate accumulation, enhanced histone lactylation, and increased Arg1 expression in bone marrow‐derived macrophages, they found no causal relationship between lactylation and Arg1 expression, with lipopolysaccharide‐induced Arg1 expression depending on autocrine‐paracrine interleukin 6 (IL‐6), IL‐6 receptor, and STAT3. The regulatory relationship between histone lactylation and Arg1 gene expression in macrophages remains controversial and requires further investigation [102]. HMGB1, released by activated macrophages, participates in the inflammatory response. Clinical studies indicate that lactate concentrations in the blood of septic patients correlate with HMGB1 levels, though the causal relationship remains unclear. Recent findings suggest that during sepsis, macrophages uptake extracellular lactate via MCTs, promoting HMGB1 lactylation and acetylation through a p300/CBP‐dependent mechanism [29]. Lactylated/acetylated HMGB1 is released from macrophages via exosomes, impairing endothelial barrier function and exacerbating sepsis progression. Utilizing extensive clinical samples, another study showed that H3K18la levels in peripheral blood mononuclear cells were significantly higher in septic shock patients than in nonseptic shock patients and healthy volunteers. H3K18la levels in septic shock patients correlated significantly with serum levels of inflammatory factors such as IL‐6 and IL‐10 [29]. This suggests that H3K18la could serve as a potential biomarker for diagnosing and predicting the severity of septic shock. In summary, high lactate concentrations in the blood of sepsis patients influence macrophage function through histone and nonhistone lactylation modifications.

### 6.2 Protein Lactylation and Tumor Immune Suppression Microenvironment

Protein lactylation is an emerging PTM that plays a crucial role in regulating various cellular processes, particularly in the context of tumor metabolism and immune evasion. Recent studies have highlighted its involvement in shaping the tumor immune suppressive microenvironment by modulating key immune and metabolic pathways. Understanding the mechanisms underlying protein lactylation in tumors may provide novel insights into cancer progression and therapeutic strategies. In this review, we systematically summarize the current research progress on protein lactylation in reshaping the tumor immune microenvironment, providing readers with a better understanding of its role in tumor immunity (Table 2). Brand et al. linked alterations in tumor glucose metabolism with immune evasion and found that increased lactate production by LDHA A in cancer cells disrupts cytokines produced by tumor‐infiltrating T cells and natural killer (NK) cells, particularly IFN‐γ. This disruption impairs tumor immune surveillance and promotes tumor growth [103]. Malignant tumor cells can evade immune system surveillance, recognition, and attack through various mechanisms, disrupting the balance between immune cells and tumor cells and continuing to grow—a phenomenon known as immune evasion [104]. PTMs of proteins are one of the critical mechanisms by which tumor cells achieve immune evasion. Xiong et al. discovered that lactate in the tumor microenvironment (TME) promotes immune suppression of tumor‐infiltrating myeloid (TIM) cells through protein lactylation. This occurs first by inducing the expression of RNA methyltransferase‐like 3 (METTL3) through H3K18 lactylation, which enhances METTL3‐mediated m6A modifications on Jak1 mRNA. The m6A‐YTHDF1 axis increases JAK1 protein translation efficiency and subsequent STAT3 phosphorylation, thus forming the METTL3/m6A/JAK1/STAT3 axis, which enhances TIMs’ immune suppressive functions and facilitates tumor immune evasion [64]. Second, lactylation of the “CCCH” zinc finger domain of METTL3 enhances its binding affinity to m6A RNA, triggering downstream reactions related to poor prognosis in colon cancer. Specific loss‐of‐function mutations in the phosphatase and tensin homolog (PTEN) gene are observed in metastatic castration‐resistant prostate cancer patients and are associated with poor tumor prognosis. PTEN loss can activate PI3K signaling, and the study found that PI3K inhibitors and anti‐PD‐1 treatments can reverse H3K18 lactylation and PD‐1‐mediated immune suppression in tumor‐associated macrophages (TAMs), thereby controlling prostate cancer growth in conjunction with androgen deprivation therapy [105]. Gu et al. [68] found that lactylation of the membrane‐associated protein Moesin enhances its interaction with transforming growth factor‐β (TGF‐β) receptors and activates the SMAD3 signaling pathway, mediating the generation of Treg cells and further promoting tumor cell immune evasion. Inhibiting Moesin lactylation can improve the efficacy of immunotherapy [106]. Wang et al. identified a unique FOXP3+ NKT‐like cell in malignant pleural effusion's “cold” TME. Single‐cell sequencing analysis revealed that FOXP3+ NKT cells highly express MCT and LDHA B, utilizing lactate to maintain their immune suppressive function. The study also found that patients with lower lactate levels had better responses to anti‐PD‐1 therapy, indicating that lactate levels directly affect lactylation levels [107]. Therefore, modulating MCTs and GPR81 receptors could control lactate levels and provide potential therapeutic benefits in cancer treatment [29]. Cyclin E2 (CCNE2) is a crucial cell cycle protein essential for liver cancer progression. Recent research has shown that nonhistone CCNE2 promotes liver cancer cell proliferation, migration, and invasion through lactylation, while NAD‐dependent deacetylase SIRT3 can remove CCNE2 lactylation, thereby regulating the cell cycle and impeding liver cancer progression. This study focuses on the effects of CCNE2 lactylation in liver cancer cells, and further research is needed to determine whether CCNE2 lactylation mediates immune suppression by immune cells or cytokines, thereby indirectly promoting liver cancer progression [57]. Lactate in the TME has been found to regulate the metabolism of immune cells, inhibiting the proliferation and function of CD8+ T cells, NK cells, and dendritic cells, thereby mediating immune evasion [21,64]. Macrophages, as key innate immune cells in the TME, are classified into M1 and M2 phenotypes based on their functions—M1 being proinflammatory and antitumor, and M2 being anti‐inflammatory and protumor. Zhang et al. found that lactate induces an increase in M2 phenotype‐associated proteins, such as vascular endothelial growth factor, in macrophages through histone lactylation, facilitating the transition of macrophages from M1 to M2 phenotypes [108]. Wang et al. discovered that the key cholesterol metabolism molecule proprotein convertase subtilisin/kexin type 9 (PCSK9) is highly expressed in colon cancer. Knockdown of PCSK9 reduced lactate levels secreted by tumor cells and macrophage migration inhibitory factor expression, further promoting M1 polarization of TAMs and inhibiting their M2 polarization [109]. Chaudagar et al. [66] found that reduced levels of phosphoinositide 3‐kinase (PI3K) in prostate cancer lead to decreased lactate synthesis in tumor cells, further inhibiting histone lactylation modifications in TAMs and enhancing their immune efficacy [110]. These findings reveal the significant role of lactate and lactylation modifications in regulating immune cell functions within the TME, offering new insights for overcoming immune suppression and improving tumor immunotherapy. Additionally, the role and mechanisms of lactylation modifications in other important cell components of the TME, such as cancer‐associated fibroblasts and endothelial cells, remain underexplored and warrant further investigation.

**TABLE 2:** Summary of the functional mechanisms of protein lactylation modifications in different tumor immune cells.

Immune cell type	Mechanisms	Target gene	Function	Ref
Tumor‐associated macrophages (TAMs)	(1) Lactylation promotes M2 polarization: Lactylation of histones (e.g., H3K18la) enhances the expression of M2‐associated genes (e.g., Arg1, CD206, IL‐10), shifting TAMs toward a protumor phenotype. (2) Enhances immunosuppressive functions: Lactylation increases TGF‐β and IL‐10 secretion, weakening T cell and NK cell activity. (3) Suppresses proinflammatory responses: Lactylation reduces IL‐12 and TNF‐α expression, impairing M1 macrophage proinflammatory functions.	H3K18la, Arg1, TGF‐β, IL‐10, STAT3	(1) Promotes tumor growth—Enhances immunosuppression (2) Facilitates angiogenesis	[109, 111, 112, 113, 114, 115]
Dendritic cells (DCs)	(1) Inhibits DC maturation: Lactylation suppresses the expression of costimulatory molecules (e.g., CD80, CD86), reducing antigen presentation capacity. (2) Promotes tolerogenic DC formation: Lactylation induces TGF‐β and IDO1 expression, leading to a tolerogenic phenotype that fails to activate T cells. (3) Reduces inflammatory signaling: Lactylation decreases IFN‐β and IL‐12 secretion, impairing CD8+ T cell activation.	CD80/CD86, IDO1, TGF‐β, IFN‐β	(1) Decreased antigen presentation (2) Reduced T cell activation (3) Enhanced immune tolerance	[116, 117, 118]
CD8+ T cells (cytotoxic T cells, CTLs)	(1) Lactylation suppresses effector function: Lactylation reduces IFN‐γ and TNF‐α expression, leading to T cell exhaustion. (2) Impairs metabolic adaptation: Lactylation decreases mitochondrial oxidative phosphorylation (OXPHOS), limiting T cell energy supply. (3) Increases immune checkpoint expression: Lactylation upregulates PD‐1 and TIM‐3, further impairing CD8+ T cell function.	IFN‐γ, TNF‐α, PD‐1, TIM‐3, OXPHOS	(1) Reduced cytotoxicity (2) Promotes T cell exhaustion (3) Enhances immune evasion	[119, 120, 121, 122, 123]
CD4+ regulatory T cells (Tregs)	(1) Lactylation enhances Treg immunosuppressive function: Lactylation modifies Foxp3 protein, stabilizing its function and enhancing Treg‐mediated suppression. (2) Promotes Treg metabolic adaptation: Lactylation regulates mTORC1 signaling, making Tregs more reliant on glycolysis for survival in the TME. (3) Suppresses antitumor immune responses: Lactylation increases IL‐10 and TGF‐β secretion, inhibiting CD8+ T cell activation.	Foxp3, IL‐10, TGF‐β, mTORC1	(1) Enhances immunosuppression (2) Promotes tumor immune evasion (3) Maintains Treg function	[124, 125, 126, 127]
Natural killer (NK) cells	(1) Lactylation suppresses NK cell activity: Lactylation reduces the expression of key cytotoxic molecules (e.g., Perforin, Granzyme B), weakening NK cell‐mediated tumor killing. (2) Impairs cytokine secretion: Lactylation decreases IFN‐γ secretion, reducing NK cell activation of antitumor immunity. (3) Promotes NK cell exhaustion: Lactylation upregulates inhibitory receptors (e.g., TIGIT, NKG2A), diminishing NK cell cytotoxicity.	Perforin, Granzyme B, IFN‐γ, TIGIT, NKG2A	(1) Reduced NK cell cytotoxicity (2) Promotes NK cell dysfunction Enhances immune evasion	[128, 129, 130]
B cells	(1) Lactylation affects antibody secretion: Lactylation regulates B cell activation, influencing IgG and IgA production. (2) Regulates Bregs (regulatory B cells): Lactylation enhances Breg secretion of IL‐10, further suppressing antitumor immune responses. (3) Inhibits antitumor B cell function: Lactylation reduces B cell antigen presentation, limiting Tfh cell activation.	IgG, IgA, IL‐10, Tfh	(1) Enhances immunosuppression (2) Affects antibody‐mediated tumor immunity (3) Promotes immune evasion	[131, 132, 133]
Myeloid‐derived suppressor cells (MDSCs)	(1) Lactylation enhances MDSC‐mediated immunosuppression: Lactylation promotes Arg1 and IDO1 expression, increasing MDSC suppression of T cells. (2) Regulates metabolic adaptation: Lactylation enhances MDSC utilization of lactate, promoting their persistence in the tumor microenvironment. (3) Inhibits T cell function: Lactylation increases reactive oxygen species (ROS) production, impairing T cell activity.	Arg1, IDO1, ROS, HIF‐1α	(1) Enhances immunosuppression (2) Promotes T cell dysfunction (3) Supports tumor growth	[134, 135, 136, 137]

Recently, research teams have used bioinformatics analysis to identify six lactylation‐related genes associated with gastric cancer prognosis, creating a “lactylation score” model. This model reveals that gastric cancer patients with high lactylation scores possess greater immune evasion potential and lower response rates to immunotherapy, enabling the prediction of patient responses to immune checkpoint inhibitors and offering more suitable treatment options [138]. Similarly, bioinformatics analysis has identified lactylation‐related genes associated with liver cancer prognosis and developed an effective prognostic model for liver cancer. Low‐risk scores indicated better responses to most targeted therapies and immunotherapies, while high‐risk scores were associated with increased sensitivity to most chemotherapeutic agents and sorafenib, suggesting that lactylation‐related gene markers could serve as biomarkers for effective clinical treatment of liver cancer [139]. In addition to macrophages activated by bacterial infection, macrophages in other inflammatory models are also regulated by lactylation. Cui et al. found that alveolar macrophages exhibit increased lactylation levels during pulmonary fibrosis. In a mouse model of pulmonary fibrosis, myofibroblasts in the lungs produce and secrete large amounts of lactate, which upregulates histone lactylation levels in alveolar macrophages. This promotes the enrichment of lactylated histones in the promoter regions of profibrotic genes such as Agr1 and platelet‐derived growth factor (PDGF), thus enhancing gene transcription. In a mouse model of dextran sulfate sodium‐induced inflammatory colitis, macrophage‐specific BCAP knockout mice exhibited exacerbated inflammation and tissue damage [79]. Further research showed that BCAP‐deficient bone marrow‐derived macrophages displayed reduced glycolytic activity, decreased intracellular lactate levels, reduced histone lactylation, and downregulated expression of genes involved in damage repair [79]. Exogenous lactate supplementation rescued this phenotype in BCAP‐deficient bone marrow‐derived macrophages, primarily by enhancing histone lactylation, thereby shifting macrophages from a proinflammatory phenotype to a reparative phenotype. Recently, another study found that lactate‐producing Saccharomyces cerevisiae significantly reduced levels of proinflammatory cytokines IL‐6 and IL‐1β in macrophages of ulcerative colitis mice, inhibiting M1 macrophage polarization and alleviating intestinal inflammation [140]. These gene expression changes might be related to lactate‐induced enhancement of H3K18la in macrophages. Additionally, elevated histone lactylation levels have been observed in TAMs. These studies suggest that the activation status of macrophages in other inflammatory conditions is also closely related to histone lactylation. However, the specific genes upregulated or downregulated by histone lactylation in different types of inflammation require further investigation. Another type of immune cell similar to macrophages in function is the microglia in the central nervous system, whose function is also regulated by histone lactylation modifications. Pan et al. discovered that in AD model mice and clinical AD patient brain tissue samples, microglia adjacent to β‐amyloid plaques exhibited significantly elevated levels of H4K12la, which epigenetically regulates the transcription of glycolytic genes, further promoting lactate production [53]. This “metabolic‐epigenetic‐metabolic” positive feedback loop stimulates microglia to release inflammatory factors, ultimately promoting AD progression.

### 6.3 Lactylation and Protein Lactylation Modification in Neurological Disorders

AD is a neurodegenerative disorder characterized by progressive cognitive impairment. Numerous studies indicate that its pathogenesis is closely associated with abnormal activation and aging of microglia [141]. Microglia possess the capability to phagocytize toxic products and release cytotoxic factors while also having antigen‐presenting functions [142]. Activated microglia secrete proinflammatory cytokines and chemokines, which can protect the central nervous system from further damage but may also adversely affect neurons and other glial cells, exacerbating damage [143]. During this process, lactate, a glycolysis product, is considered a significant mediator that directly enhances the release of proinflammatory cytokines from microglia. Pan et al. observed that the levels of PanKla and H4K12la were significantly elevated in the brain tissue of AD patients and 5XFAD transgenic AD model mice. Notably, H4K12la was enriched in the promoter regions of glycolysis genes and promoted the transcription of glycolysis‐related genes. Additionally, the specific deletion of pyruvate kinase M2 (PKM2) in microglia reduced Aβ accumulation in 5XFAD mice and improved their spatial learning and memory abilities [53]. This study elucidated a positive feedback loop involving glycolysis, H4K12la, and PKM2, which drives the activation of proinflammatory microglia, thereby exacerbating metabolic disorders and microglial dysfunction in AD patients. Conversely, disrupting this loop diminished microglial activation and neuroinflammation. Therefore, targeting the glycolysis‐H4K12la‐PKM2 feedback loop could be an effective strategy for treating AD [53]. Research indicates that metabolically active aging microglia further threaten aging neurons, promoting age‐related neurodegenerative changes. Wei et al. [30] found that both PanKla and H4K12la were elevated in naturally aging microglia and hippocampal tissues, with histone lactylation being more pronounced [144]. They identified that all histone lysine lactylation sites were elevated in aging microglia and hippocampi of naturally aging and AD mice, with H3K18la being significantly upregulated. Increased lactate levels promoted H3K18 lactylation in aging microglia and hippocampi, while LDHA inhibitors reduced lactate levels, leading to a significant decrease in histone lysine lactylation [144]. This further validated that elevated histone PanKla in aging microglia is related to increased lactate concentration. Integrating ChIP‐qPCR and RNA‐seq data, they confirmed the mechanism by which H3K18la impacts brain aging and AD. They found that H3K18la increased its binding to the Rela (p65) and NFκB1 (p50) promoters, leading to the activation of the NFκB signaling pathway and promoting the generation of age‐related secretory phenotype (SASP) components IL‐6 and IL‐8, further influencing brain aging and AD phenotypes [145]. The results suggest that the H3K18la/NFκB signaling axis targets neuroinflammation, exacerbating brain aging and AD pathology. This study also hints at an H3K18la/NFκB axis/SASP positive feedback loop that drives the pathogenesis of brain aging and AD. H4K12la expression is increased in the hippocampi and cortices of AD model mice, while H3K18la is significantly elevated in the hippocampi of naturally aging and AD mice, but remains unchanged in cortical tissues. This indicates that H4K12la and H3K18la exhibit differential expression patterns and have distinct biological functions and downstream targets in AD pathogenesis [146]. Therapeutic strategies targeting H4K12la and H3K18la may help in intervening AD progression, particularly by modulating glycolysis and inflammation‐related signaling pathways, potentially offering more treatment options. However, further research is needed to validate these findings and develop specific therapeutic approaches to more effectively address the complex disease mechanisms of AD. Hagihara et al. [28] found that lactate‐mediated lysine lactylation is prevalent in brain cells. Using proteomics, they identified 63 candidate lactylated proteins in the prefrontal cortex of an SDS mouse model. Increased lactate levels due to neuronal excitation promoted the lactylation of histone H1 lysine in the PFC, and the expression of the neuronal activity marker c‐Fos also increased in the PFC. Elevated H1 lactylation was associated with increased behavioral anxiety and reduced social interaction in SDS mice [147]. This study confirmed that lactylation is present in the brain and may play a significant role in neuronal activity. Recent studies have found that IDH3β is downregulated in patients with AD and AD transgenic mice. Knockout of IDH3β induces oxidative‐phosphorylation uncoupling, leading to reduced energy production and lactate accumulation. The increased lactate promotes histone lactylation, which enhances the expression of paired box gene 6 (PAX6). Elevated PAX6, in turn, suppresses IDH3β expression, resulting in tau hyperphosphorylation, synaptic damage, and learning and memory impairments, forming a vicious cycle that exacerbates the progression of AD [148].

Acute ischemic stroke (AIS) is a neurovascular disorder caused by arterial occlusion leading to inadequate blood supply. Timely restoration of blood flow is currently the primary treatment for AIS [149]. However, the reperfusion process can further exacerbate neuronal injury in the ischemic area, ultimately leading to neuronal apoptosis or necrosis and resulting in cerebral ischemia reperfusion injury (CIRI). The pathogenesis of CIRI involves multiple factors, including cellular energy deficiency, glutamate excitotoxicity, oxidative stress, cell apoptosis, Ca2+ overload, acidosis, inflammatory responses, blood‐brain barrier disruption, excessive free radical production, mitochondrial dysfunction, and nitrosative stress [150]. Research has confirmed that cellular acidosis in ischemic regions leads to insufficient energy supply, with significant lactate accumulation causing acidosis and subsequent metabolic imbalance, interfering with intracellular protein synthesis. Moreover, acidosis can mediate neuronal excitotoxicity by affecting Na+ channel kinetics [151]. Yao et al. measured the lactylation levels of brain endothelial proteins in CIRI rats using pan anti‐Kla antibodies [152]. The results showed that Kla levels were significantly higher in CIRI rats compared to healthy controls. Subsequently, combining 4D label‐free quantitative proteomics with lactylation‐specific proteomics analysis, they assessed Kla sites in cortical proteins of CIRI rats, confirming that lactylation is involved in the pathogenesis of CIRI. This finding provides valuable insights into the pathological mechanisms of CIRI. Studies have indicated that elevated Ca2+ concentrations lead to increased active calmodulin proteins, including CaM (calmodulin M) and serotonin, norepinephrine, and so on, which are formed after binding with Ca2+ [152]. These substances can cause intense vasospasm and contraction, leading to decreased perfusion in post‐ischemic organs and further exacerbating cellular toxicity. In hypoxic conditions where the respiratory chain is obstructed, the adenine nucleotide translocator (ANT) transports ADP to the cytoplasm and ATP to the mitochondrial matrix. This ADP/ATP translocation triggers severe metabolic disorders. On the other hand, voltage‐dependent anion channel protein 1 (Vdac1), a common channel protein in the mitochondrial outer membrane, provides a water channel from the cytoplasm to the mitochondrial outer membrane [153]. This allows Ca2+ and matrix components with a molecular weight of less than 1500 to easily traverse the mitochondrial membrane. Yao et al. used mass spectrometry and other techniques to detect two lactylated ANT variants in CIRI rats, including Slc25a4 and Slc25a5. In contrast, lactylated Vdac1 was detected in healthy control rats [154]. These results indicate that lactylation modifications are crucial for Ca2+‐induced CIRI. Subsequent in vivo experiments in rat brain endothelial tissues showed that Vdac1 lactylation levels decreased in CIRI rats compared to the control group, affecting mitochondrial apoptosis pathways and mediating neuronal death. However, it is noteworthy that due to the lack of Slc25a4 and Slc25a5 antibodies, the roles of Slc25a4/Slc25a5 lactylation could not be further verified in this experiment. Heat shock protein A12A (HSPA12A) is an atypical member of the HSP70 family, reported to alleviate brain function damage in ischemic stroke and promote long‐term functional recovery. It can also enhance angiogenesis and aid in recovery after myocardial infarction [155]. Recently, studies reported that liver cell HSPA12A inhibits macrophage chemotaxis and activation by suppressing lactate‐mediated HMGB1 lactylation and secretion, thereby reducing liver ischemia/reperfusion injury [156].

### 6.4 Lactylation and Metabolic Diseases

Metabolic diseases, such as diabetes, obesity, non‐alcoholic fatty liver disease (NAFLD), and cardiovascular diseases, are closely associated with lactate metabolism dysregulation [157]. Studies have shown that lactate, as a key metabolic intermediate, is not merely a byproduct of glycolysis but also participates in regulating various metabolic processes through protein lactylation. In diabetes, lactate accumulation is commonly observed in the pathological states of insulin resistance and hyperglycemia [158]. Lactylation may influence insulin sensitivity by modulating proteins related to insulin signaling pathways (such as the PI3K/AKT pathway) or inflammatory factors (such as the NF‐κB signaling pathway). In obesity‐related chronic inflammation, the level of lactylation modification in macrophages within adipose tissue is elevated [159]. Lactylation may promote the release of inflammatory factors, exacerbating metabolic dysregulation. During the pathological progression of NAFLD, lactylation modifications affect lipid metabolism in hepatocytes by regulating the function of key enzymes such as fatty acid synthase and acetyl‐CoA carboxylase, thereby influencing fat accumulation and liver inflammation [160].

### 6.5 Lactylation and Cardiovascular Diseases

Atherosclerosis is an inflammation‐associated disease wherein macrophages play a crucial role in regulating the progression of atherosclerotic plaques [161, 162]. As previously mentioned, macrophages exhibit significant plasticity and can polarize in response to different environmental signals. In atherosclerosis, M1 macrophages promote plaque rupture, while M2 macrophages can protect plaques and facilitate inflammation resolution. Zhang et al. have discovered that histones in macrophages can undergo lactylation, inducing the conversion of macrophages from M1 to M2 types [163]. Whether these lactylated histones also play significant roles in atherosclerosis warrants further investigation. Myocardial infarction triggers a complex inflammatory cascade. Following myocardial infarction, circulating monocytes undergo metabolic reprogramming, with an increase in glycolytic flux leading to elevated lactate levels. This increase in lactate promotes higher levels of histone lactylation. Recent research by Professor Yu Bo's team has found that post‐myocardial infarction, IL‐1β recruits HAT 5 (GCN5) to mediate H3K18 lactylation in circulating monocytes, thereby regulating the expression of VEGF‐α, IL‐10, and LRG1 genes involved in angiogenesis and inflammatory repair [12]. A recent interesting study utilized lactylation modification proteomics to reveal the lactylation landscape in cardiac tissue from heart failure mice. The study found a significant decrease in lactate levels in the heart tissue of the heart failure mice, which further led to a marked reduction in the lactylation modification level of α‐MHC K1897 and the interaction between α‐MHC and Titin, thereby promoting heart failure in the mice [164]. A concurrent study found that NR4A3 promotes lactate production by enhancing glycolytic activity, further mediating histone lactylation to upregulate Phospho1 and promote arterial calcification. This study reveals that NR4A3‐driven histone lactylation is a novel mechanism regulating arterial calcification [165].

### 6.6 Lactylation and Cancer Progression

In the 1920s, Otto Warburg and his team discovered the characteristic of “aerobic glycolysis” in tumors, known as the Warburg effect, revealing that tumor tissues require more glucose than surrounding tissues and convert glucose into lactate through glycolysis [166]. Lactate is further oxidized to produce a large amount of ATP, providing energy for tumor cells. Given the prevalent glycolytic metabolism in tumors and the epigenetic modifications induced by high levels of lactate inside and outside tumors, lactylation has significant research potential in tumors. Current views suggest that histone and nonhistone lactylation in tumor cells can regulate intracellular signal transduction, gene expression, and protein function, thereby affecting tumor proliferation, differentiation, and adaptability to the environment [167]. Recent studies have also detected protein lactylation in various tumor cells, indicating that lactylation might become a new target for tumor therapy. Here, we summarize the functions and mechanisms of protein lactylation in the progression of various human tumors (Table 3).

**TABLE 3:** Summary of the functions, target genes, and signaling pathways of lactylation in different cancers.

Types of cancer	Lactylation site	Target gene	Function	Ref
Acute myeloid leukemia	H3K18, H4K5, H4K8, H4K12	STAT5‐lactylation‐PD‐L1	Promoted E3BP nuclear translocation and histone lactylation, inducing PD‐L1 transcription.	[122]
Bladder cancer	H3K18	circXRN2‐LATS1‐hippo‐lactylation‐LCN2	Suppresses tumor progression driven by H3K18 lactylation by activating the Hippo signaling pathway	[168]
Cervical cancer	K45 on G6PD	G6PD(delactylation)‐GP6D(dimer)	Activates the pentose phosphate pathway to promote cervical cancer cell proliferation	[169]
Cervical cancer	K172 on DCBLD1	DCBLD1‐G6PD‐pentose phosphate pathway	Lactate‐induced DCBLD1 lactylation stabilized DCBLD1 expression	[170]
Clear cell renal cell carcinoma	H3K18	Histone lactylation‐PDGFRβ signaling positive feedback loop	Inactive VHL‐triggered histone lactylation contributes to ccRCC progression	[90]
Colorectal cancer	H3K18	GPR37‐LATS1‐YP1‐lactylation‐CXCL1/CXCL5	Promoting LDHA expression and glycolysis	[171]
Colorectal cancer	H4K12	SMC4‐diapuse like state‐glycolysis enzymes‐lactylation	Increase ABC transporter expression via histone lactylation, rendering tumor cells insensitive to chemotherapy	[55]
Colorectal cancer	H3K18	Lactylation‐RUBCNL	Facilitating autophagosome maturation	[11]
Colorectal cancer	Pan‐lactylation	PCSK9‐lactylation‐M2 polarization	Induce colon cancer cell epithelial‐mesenchymal transition (EMT) process and activated PI3K/AKT signaling	[109]
Colorectal cancer	H3K18	Lactylation‐RARγ‐NF‐kB‐IL‐6	Prohibit RARγ gene transcription in macrophages	[112]
Colorectal cancer	K408 on eEF1A2	KAT8‐eEF1A2	Inhibited CRC tumor growth	[172]
Endometrial carcinoma	H3K18	Kla‐USP39‐PGK1‐PI3K/AKT/HIF‐1α signaling pathway	Inhibit the proliferation and migration ability, induce apoptosis	[173]
GC	K90 on YAP, K108 on TEAD1	Hippo pathway	Activate the YAP‐TEAD complex and promote gastric cancer cell proliferation	[45]
Glioblastoma	H3K18	Lactylation‐CD39, CD73, CCR8	Increasing regulatory T (Treg) cells infiltration	[174]
Glioblastoma	H3K18	Lactate‐lactylation‐MAP4K4‐JNK pathway	Promotion of self‐renewal in GBM cells	[175]
Liver cancer	K348 on CCNE2	SIRT3‐CCNE2	Suppress HCC development	[57]
Liver cancer	K72 on MOESIN	MOESIN‐TGFb pathway‐SMAD‐FOXP3	Increases antitumor immunity, and decreases tumor growth	[106]
Ocular melanoma	H3K18	Lactylation‐YTHDF2 ‐YP53/PER1	Contributes to tumorigenesis by facilitating YTHDF2 expression	[93]
Pancreatic adenocarcinoma	K128 on NMNAT1	NMNAT1‐NAD salvage pathway	Sustain nuclear NAD+ salvage pathway and promote survival of pancreatic adenocarcinoma	[176]
Prostate cancer	H3K18	Lactylation‐HIFa‐sema3A	Blocked lactate‐induced angiogenesis by restricting histone lactylation	[177]
Prostate cancer	K215 and K224 on CNPY3	CNPY3‐pyroptosis	Promote lysosome rupture for triggering pyroptosis	[178]

#### 6.6.1 Lung Cancer

The histological forms of lung cancer mainly include NSCLC and small cell lung cancer (SCLC), accounting for 85% and 15%, respectively. Studies have found that treating NSCLC cells with lactate increases histone lactylation levels, significantly enhancing tumor cell growth and proliferation [179]. Furthermore, lactate can downregulate the mRNA levels of glycolytic enzymes such as hexokinase‐1 (HK‐1) and pyruvate kinase isozyme, and upregulate the mRNA levels of tricarboxylic acid cycle enzymes such as succinate dehydrogenase and isocitrate dehydrogenase 3 noncatalytic subunit gamma (IDH3G). Chromatin immunoprecipitation assays have also observed increased histone lactylation levels in the promoters of HK‐1 and IDH3G. These results suggest that lactate promotes NSCLC development, at least partly through histone lactylation‐mediated gene expression, although the mechanisms of lactylation regulation of gene transcription require further elucidation [180]. Another study found that elevated histone lactylation levels in lung adenocarcinoma cells significantly reduced the expression of the solute carrier family 25 member 29 (SLC25A29) gene, promoting lung adenocarcinoma cell proliferation and migration [181]. Subsequent research demonstrated that hypoxia‐induced lactylation of the transcription factor SOX9 enhances glycolysis, promoting the stemness, migration, and invasion of NSCLC cells [182]. However, it remains unclear whether lactylation is associated with SCLC.

#### 6.6.2 Liver Cancer

Lactylation can promote the development of HCC. Pan et al. reported that DML, a triterpene antitumor compound, inhibits HCC development and progression by interfering with histone lactylation, providing a theoretical basis for DML as a potential adjunctive therapy for HCC. Subsequent research by this team found that royal jelly acid also inhibits HCC cell proliferation and migration and promotes apoptosis by downregulating histone lactylation [73]. Both studies confirmed that inhibiting histone lactylation can hinder HCC progression, but the mechanisms by which lactylation intervenes in HCC development and progression are not deeply explored. Another study showed that cyclin E2 (CCNE2) lactylation promotes HCC growth, while deacetylase can downregulate CCNE2 lactylation levels to inhibit HCC cell proliferation, migration, and invasion [57]. This study links nonhistone lactylation with liver tumors, providing a new direction for research on lactylation and tumors. Centromeric proteins (CENP) are potential markers related to tumor development, and research has found that lactylation of CENPA at lysine 124 (K124) promotes its activation and, through interaction with the transcription factor YY1, promotes HCC proliferation. Additionally, lactylation‐related gene characteristics can effectively predict HCC prognosis and treatment response, providing important clues for clinically assessing HCC treatment efficacy and prognosis. Recently, a multiomics study revealed the global lactylation modification landscape in a cohort of hepatitis B virus‐associated HCC, integrating proteomics and lactylation modification omics to identify 9275 lactylation modification sites, with 9256 sites located on nonhistone proteins. This indicates that lactylation modification is a common modification beyond histones and transcriptional regulation. Notably, lactylation preferentially affects enzymes related to metabolic pathways, including the tricarboxylic acid cycle, and carbohydrate, amino acid, fatty acid, and nucleotide metabolism. Further research showed that lactylation at K28 of adenylate kinase 2 (AK2) inhibits its function, promoting HCC cell proliferation and metastasis [36].

#### 6.6.3 Colorectal Cancer

Research on lactylation in colorectal cancer (CRC) is relatively abundant, demonstrating that lactylation can promote the growth, proliferation, and migration of CRC cells while diminishing the effectiveness of drug treatments [11]. Lactylation can accelerate tumor cell growth and proliferation by promoting immune evasion in colorectal tumors. Studies have shown that lactate derived from colorectal tumors directly mediates the lactylation of the methyltransferase‐like METTL3 zinc finger domain, which enhances the binding and catalytic activity of METTL3 on Janus kinase 1 (JAK1) mRNA, facilitating m6A modification [64, 183]. This modification increases the translation efficiency of JAK1 mRNA and enhances the phosphorylation level of downstream signal transducer and activator of transcription STAT3. Through the downstream JAK‐STAT signaling pathway, METTL3 lactylation promotes the expression of immunosuppressive effector molecules such as interleukin (IL‐6, IL‐10, and inducible nitric oxide synthase), ultimately promoting tumor immune evasion and accelerating tumor growth and proliferation [64]. This research also provides a theoretical basis for novel immunotherapeutic strategies involving METTL3 inhibitors for CRC.

Other studies have indicated that the expression of proprotein convertase subtilisin/kexin type 9 (PCSK9) is upregulated in colon cancer tissues. This upregulation induces M2 polarization of TAMs by elevating lactate and lactylation levels, thus promoting immune evasion and accelerating the growth and proliferation of colon cancer cells. Inhibiting PCSK9 expression yields opposite results [109]. Lactylation can directly promote the growth and proliferation of colorectal tumor cells by regulating different signaling pathways. For instance, hypoxia‐induced glycolysis promotes β‐catenin lactylation in CRC tissues, which, in turn, promotes tumor growth and proliferation by regulating the Wnt signaling pathway [184]. Aldolase B (ALDOB)‐mediated lactylation promotes the growth and proliferation of CRC cells by increasing the stability of carcinoembryonic antigen‐related cell adhesion molecule 6 (CEACAM6), which is believed to enhance the growth and proliferation of CRC cells [185].

Recent studies have demonstrated that lactylation drives the downregulation of retinoic acid receptor gamma (RARγ) in TAMs, thereby enhancing IL‐6 levels in the TME and promoting tumor growth and proliferation through the activation of STAT3 signaling in colorectal tumor cells. This research also found that demethoxycurcumin could exert effective antitumor effects by directly binding to RARγ to inhibit its downstream signaling, although the role of lactylation in this process was not further explored [186]. Lactylation can promote the invasion and migration of colorectal tumor cells. Research has shown that histone lactylation at the H4K8 site significantly increases in colorectal tumor cells HCT116 treated with lipopolysaccharide, and this increase in histone lactylation regulates the expression of long intergenic nonprotein coding RNA 152 (LINC00152), thereby promoting tumor cell invasion and migration [187]. Lactylation can also promote liver metastasis of colorectal tumors. Studies have found that GPR37 expression is significantly increased in liver metastatic tissues of rectal cancer, accompanied by poor prognosis. The mechanism may involve GPR37 activating the Hippo signaling pathway, which promotes glycolysis leading to increased histone lactylation, further causing the upregulation of CXC chemokine ligand (CXCL) 1 and CXCL5, thereby mediating liver metastasis of rectal cancer [171]. Lactylation can increase the resistance of colorectal tumors to chemotherapy, reducing their sensitivity to chemotherapy. The weakened function of structural maintenance of chromosome protein 4 promotes the expression of glycolytic enzymes, elevating lactate levels. The accompanying histone lactylation increases the expression of ATP‐binding cassette (ABC) transporters, reducing the sensitivity of CRC cells to chemotherapy. Histone lactylation can promote the expression of autophagy enhancer protein RUBCNL, thereby enhancing the resistance of CRC to bevacizumab treatment [11]. Recent research has found that ALDOB‐mediated lysine lactylation increases the chemotherapy resistance of CRC cells to 5‐fluorouracil by stabilizing CEACAM6 expression levels [185]. Recent studies have shown that the overall Kla abundance distribution in CRC is negatively correlated with patient prognosis. In CRC, KAT8, identified as a lysine acetyltransferase and a lactyltransferase, is responsible for transferring lactate to various proteins. The loss of KAT8 inhibits CRC tumor growth, especially in high‐lactate TMEs. As a lactyltransferase, KAT8 directly lactylates eEF1A2 K408, enhancing translation elongation rate and protein synthesis, thereby promoting tumorigenesis [172].

#### 6.6.4 Breast Cancer

Lactate metabolism in breast cancer cells promotes elevated histone lactylation levels, further regulating the high expression of the oncogenic transcription factor c‐Myc. c‐Myc drives the alternative splicing of genes MDM4 and Bcl‐x in breast cancer cells by transcriptionally upregulating serine/arginine‐rich splicing factor 10 (SRSF10), thereby accelerating breast cancer progression [188]. Conversely, inhibiting glycolysis rates, reducing lactate levels and lactylation, can successfully inhibit the c‐Myc‐SRSF10 axis and impede breast cancer progression. This study is the first to reveal the regulatory role of lactylation on oncogenes in breast cancer and suggests a potential therapeutic strategy to prevent glycolytic cancers by reducing lactate levels and lactylation through glycolysis inhibition. However, this study did not quantify lactyl‐CoA, thus failing to further elucidate the interaction between lactylation‐mediated metabolic changes and epigenetics [188]. Ziziphus jujuba, a type of jujube saponin, can enhance caspase‐3 activity, induce mitochondrial apoptosis, and ultimately promote cancer cell apoptosis while modulating lactylation and 2‐hydroxyisobutyrylation levels to suppress breast cancer progression [189].

#### 6.6.5 Melanoma

Research has shown that histone lactylation is significantly elevated in ocular melanoma, promoting tumor growth. The mechanism involves lactylation upregulating the expression of YTH domain family protein 2 (YTHDF2), which recognizes N6‐methyladenosine (m6A) modification sites on period circadian regulator 1 (PER1) and tumor protein p53 (TP53) mRNAs, regulating their degradation [93]. This study is the first to reveal the link between lactylation and tumors, providing a new therapeutic target for ocular melanoma and associating lactylation with RNA modification, offering new insights into epigenetic regulation during tumorigenesis. Since this study only analyzed ocular melanoma, it remains uncertain whether lactylation affects melanomas originating from the skin and other sites. Another study found that increased lactate levels in uveal melanoma cells lead to elevated lactylation levels, thereby inhibiting melanoma cell proliferation. Conversely, inhibiting lactate uptake promotes melanoma cell growth and proliferation, potentially related to increased MCT1 expression caused by elevated lactate levels. These two studies using the same human uveal melanoma cells yield different results, suggesting diverse and complex regulatory targets of lactylation in ocular melanoma [190].

#### 6.6.6 Prostate Cancer

Research has observed that in prostate‐specific PTEN/p53 gene‐deficient mice, an animal model of invasive and variable prostate cancer, various types of combination therapies extend the survival of prostate cancer mice. This extension is accompanied by reduced levels of histone lactylation and enhanced phagocytic activity of activated TAMs, suggesting that lactylation may promote prostate tumor cell growth by inducing M2 polarization of TAMs. Another study indicates that evodiamine can inhibit prostate cancer growth by reducing lactylation of hypoxia‐inducible factor 1 alpha (HIF1α) in prostate cancer cells, a finding corroborated by animal experiments. The mechanism may involve HIF1α lactylation enhancing the transcription of the KIAA1199 gene, thereby promoting angiogenesis and tumor development in prostate cancer [191]. Additionally, another study has found that the Num/Par‐kin pathway plays a critical role in maintaining mitochondrial quality and membrane potential, serving as a key metabolic switch. When the Num/Par‐kin pathway is disrupted, neuroendocrine prostate cancer cells exhibit numerous fragmented mitochondria with low membrane potential and induce metabolic reprogramming. This reprogramming accelerates glycolysis, leading to high levels of histone lactylation and increased transcription of neuroendocrine‐related genes [192].

#### 6.6.7 Kidney Cancer

ccRCC is associated with poor prognosis, with a 5‐year survival rate below 10%. Approximately 90% of ccRCC patients have mutations in the von Hippel−Lindau (VHL) tumor suppressor gene, representing a significant molecular pathological change in ccRCC [90]. In ccRCC, inactive VHL is positively correlated with histone lactylation modifications, and high levels of histone lactylation are indicative of poor prognosis. Research by Yang et al. found that inactive VHL‐induced H3K18 lactylation can activate the transcription of platelet‐derived growth factor receptor β (PDGFRβ). Conversely, PDGFRβ can induce H3K18 lactylation, creating a carcinogenic H3K18 lactylation‐PDGFRβ positive feedback loop. Targeting this loop may inhibit the proliferation and migration of ccRCC cell lines 786‐O and A498, suggesting that targeting this loop could be a novel strategy for treating ccRCC [90].

#### 6.6.8 Other Cancers

Lactylation has also been implicated in various other cancers. For instance, STAT5 promotes the expression of programmed death ligand‐1 in acute myeloid leukemia cells through increased histone lactylation, driving immune suppression in acute myeloid leukemia [122]. The circXRN2 gene can inhibit tumor progression driven by histone lactylation by activating the Hippo signaling pathway in human bladder cancer cells. Loss of fructose‐1,6‐bisphosphatase 1 expression can inhibit bladder cancer cell proliferation, migration, and invasion by suppressing lactylation at the H3K18 site [168]. BRAFV600 gene‐reconstructed cells show that lactylation can promote the proliferation of undifferentiated thyroid cancer. Lactylation of nucleolar and spindle‐associated protein 1 (NUSAP1) inhibits its degradation, upregulating NUSAP1 expression and promoting the metastasis of pancreatic ductal adenocarcinoma [193]. The oncogene DCBLD1 overexpresses through lactylation, subsequently promoting cervical cancer progression via activation of the pentose phosphate pathway. However, other studies have confirmed that lactylation can have anticancer effects, such as nonhistone METTL16 lactylation promoting copper‐induced cell death in gastric cancer cells through m6A modification on the ferritin reductase 1 mRNA, thereby inhibiting gastric cancer progression. Inhibition of glucose‐6‐phosphate dehydrogenase lactylation can promote cervical cancer cell proliferation by activating the pentose phosphate pathway, suggesting that lactylation may inhibit cervical cancer cell proliferation [37]. These studies indicate the diverse regulatory roles of lactylation in tumor development. Elevated histone lactylation is observed in undifferentiated thyroid cancer, where the oncogene BRAFV600E increases glycolysis rates, altering histone lactylation and causing dysregulated expression of cell cycle‐related genes driven by H4K12 lactylation [37].

## 7 Targeting Lactylation Modifications for Cancer Therapy

Research on lactylation modifications and related enzymes suggests that targeting lactylation modifications represents a novel approach to inhibit tumor progression and enhance antitumor effects, offering new targets for the development of anticancer drugs [8]. Current studies primarily focus on inhibitors of proteins involved in lactate metabolism and transport, as well as those in the lactylation modification processes. Here, we summarize the potential approaches for targeting protein lactylation in cancer therapy (Table 4).

**TABLE 4:** Applications of targeting lactylation in cancer therapy.

Therapeutic strategy	Mechanisms	Function	Targets	Experimental progress	Ref
Lactate metabolism inhibitors	(1) Inhibiting lactate production: Suppressing lactate dehydrogenase A (LDHA) or pyruvate dehydrogenase (PDH) to reduce intracellular lactate levels and decrease lactylation modifications. (2) Reducing lactate accumulation: Inhibiting monocarboxylate transporters (MCTs) to block lactate export, lowering tumor microenvironment (TME) lactate concentration and reducing immunosuppression.	(1) Restores CD8+ T cell cytotoxic function (2) Reduces immunosuppressive activity of TAMs and Tregs (3) Enhances antitumor immunity	LDHA, PDH, MCT1/4, HIF‐1α	(1) R LDHA inhibitor FX11 has demonstrated antitumor effects in prostate and breast cancer models. (2) R MCT1/4 inhibitor AZD3965 is being evaluated in a Phase I clinical trial for lymphoma.	[194, 195, 196, 197]
Targeting lactylation removal enzymes	(1) Enhancing delactylation enzyme activity: Activating histone deacetylases (HDACs) or sirtuin (SIRT) family proteins to increase lactylation removal. (2) Inhibiting lactylation‐promoting enzymes: Suppressing the activity of lactylation‐associated enzymes (e.g., P300/CBP) to prevent protein lactylation.	(1) Reduces immunosuppressive functions of TAMs and Tregs (2) Enhances tumor antigen presentation and T cell activity	SIRT3/6, HDACs, P300/CBP	SIRT3, as a lactylation removal enzyme, suppresses immune evasion in breast cancer cells and enhances PD‐1 blockade efficacy in melanoma models.	[198, 199, 200]
Combination with immune checkpoint inhibitors (ICIs)	(1) Reducing PD‐1/PD‐L1‐dependent immunosuppression: Decreasing lactylation modifications to alleviate T cell exhaustion and enhance ICI efficacy. (2) Enhancing T cell metabolic adaptation: Improving mitochondrial function in T cells by reducing lactylation, boosting antitumor immune responses.	(1) Increases response rates to ICIs (PD‐1/PD‐L1, CTLA‐4) (2) Improves T cell function and overcomes exhaustion	PD‐1/PD‐L1, CTLA‐4, TIM‐3	LDHA inhibitors combined with PD‐1 blockade show synergistic antitumor effects in NSCLC and melanoma models.	[201, 202, 203]
Targeting lactate clearance in the TME	(1) Promoting lactate clearance: Enhancing lactate metabolism (e.g., boosting lactate oxidation or conversion to glucose) to reduce lactate levels in the TME. (2) Regulating immune cell adaptation: Decreasing TME acidification to enhance CD8+ T cell and NK cell function while suppressing TAMs and Tregs.	(1) Enhances immune activation in the TME (2) Reduces immunosuppressive effects of TAMs and MDSCs (3) Improves CAR‐T cell efficacy	LDHB, MCTs, AMPK	Lactate oxidation enhancers (DCA) improve immune checkpoint therapy in melanoma and colorectal cancer models.	[204, 205]
Lactylation biomarkers for precision medicine	(1) Lactylation as a predictive biomarker for ICI response: High lactylation levels correlate with ICI resistance, and lactylation biomarkers can help predict patient response. (2) Targeting lactylation modifications for personalized treatment: Utilizing lactylation levels to guide the selection of metabolic or immunotherapy strategies.	(1) Enhances personalized immunotherapy strategies (2) Improves precision medicine in cancer treatment	H3K18la, LDHA, PD‐L1	H3K18la is identified as a lactylation biomarker in breast and gastric cancer, closely associated with prognosis.	[206, 207]

### 7.1 Inhibitors of Lactic Acid Metabolism

Inhibiting the activity of key enzymes in intracellular lactic acid metabolism can effectively suppress lactylation modifications by reducing lactic acid levels. LDHA A is a crucial enzyme in the glycolytic conversion of pyruvate to lactate, and its upregulation is an indicator of poor prognosis in various malignant tumors [8]. Several effective LDH inhibitors have been identified; for instance, Oxamate acts as a competitive LDHA inhibitor by competing with pyruvate, thereby inhibiting tumor cell proliferation [208]. NADH‐competitive LDHA inhibitors, including Gossypol (also known as AT‐101), FX11 (3,3'‐dihydroxy‐6‐methyl‐7‐(phenylmethyl)‐4‐propyl‐naphthalene‐1‐carboxylic acid), and Quinolinesulfonamide, have also been demonstrated to inhibit tumor cell proliferation. Galloflavin directly binds and inhibits LDHA activity, reducing lactate production and inducing apoptosis in tumor cells [209]. However, the nontarget effects and complex interactions of LDHA inhibitors with other cellular components limit their clinical development due to potential uncontrollable side effects. Dichloroacetate is an oral small‐molecule drug that inhibits pyruvate dehydrogenase kinase and promotes glucose oxidation in glycolysis, thereby activating mitochondrial apoptosis, inhibiting tumor cell proliferation, and showing efficacy in glioblastoma and advanced head and neck squamous cell carcinoma [210]. Nevertheless, lactic acid metabolism‐related inhibitors not only affect intracellular lactylation levels but also have broader and more complex impacts on cellular metabolic reactions. Therefore, improving the specificity of these inhibitors, reducing their side effects, and analyzing their inhibitory effects on specific lactylation modifications are essential for developing targeted treatments based on lactylation modifications.

### 7.2 Inhibitors of Lactic Acid Transport Proteins

In addition to its intracellular production, lactate can be shuttled and transported between cells via MCTs, further participating in various cellular physiological and pathological processes. Tumor cell lactate metabolism relies on lactate efflux mediated by MCT4 and lactate influx mediated by MCT1, making targeting MCTs potentially beneficial for treating chemotherapy‐resistant cancers [211]. Various MCT inhibitors have been identified, including α‐cyano‐4‐hydroxycinnamate (CHC), Phloretin, p‐chloromercuribenzenesulphonate, 4,4'‐diisothiocyanatostilbene‐2,2'‐disulphonic acid, Lonidamine, and Quercetin, which show therapeutic potential against multiple tumors [212]. Among them, Lonidamine, a triterpenoid antitumor compound, can inhibit HCC stem cell carcinogenicity by suppressing lactylation modifications at histone H3K9 and H3K56, and has been studied in combination with standard chemotherapy for solid tumors [213]. Additionally, some newly discovered MCT small‐molecule inhibitors exhibit higher selectivity. AR‐C155858, AZD3965, and BAY‐8002 have shown potent MCT1 inhibition and immunomodulatory activity, with a completed Phase I clinical trial confirming AZD3965's safety and efficacy in patients with advanced solid malignancies and lymphomas [82]. Future research should further elucidate the specific roles of MCTs in different cancers and lactylation modifications, as well as their structural basis and regulatory mechanisms, to provide crucial evidence for developing more specific and targeted MCT inhibitors for clinical use.

### 7.3 Targeting Lactylation Modification in Combination With Tumor Immunotherapy

Targeted inhibition of lactylation modifications combined with immune checkpoint inhibitors, such as PD‐1/PD‐L1 inhibitors, can enhance antitumor immune responses. For example, lactylation modifications play a crucial role in cancer immunotherapy. The efficacy of programmed cell death protein 1 (PD‐1) blockade immunotherapy is determined by the competition activated by PD‐1‐expressing CD8+ T cells and Treg cells within the TME [214]. Gu et al. found that lactate promotes tumor progression by modulating lactylation modifications of MOESIN in Treg cells, and that lactylation levels are lower in Treg cells from patients with effective PD‐1 antibody therapy for HCC [106]. Kumagai et al. [86] observed that in highly glycolytic malignant tumors, Treg cells rapidly absorb lactate from the TME via MCT1, stimulating the nuclear factor of activated T cells 1 (NFAT1) to enter the nucleus and increase PD‐1 expression, while PD‐1 expression in effector T cells is suppressed, leading to therapeutic failure [215]. Lactate can also induce immune resistance by promoting the expression of programmed cell death ligand 1 (PD‐L1) on macrophages and neutrophils. Weng et al. reported that targeting the MCT1/miR34a/IL‐6/IL‐6R signaling axis can inhibit M2 polarization of macrophages in triple‐negative breast cancer [216]. Preclinical studies suggest that combining MCT1 inhibitors such as AZD3965 with anti‐PD‐1 therapies can reduce lactate release into the TME and enhance antitumor immunity. Additionally, combining anti‐PD‐1 drugs with LDHA inhibitors has shown stronger antitumor effects than anti‐PD‐1 drugs alone [216]. Additionally, current interventions targeting lactylation modifications mainly focus on inhibiting lactate production, transport, and signaling, with their specificity and effectiveness for lactylation modifications requiring improvement. Continued exploration and identification of specific proteins involved in lactylation modifications are crucial for targeting lactylation and providing new targets for cancer treatment.

### 7.4 The Limitations of Targeting Protein Lactylation for Cancer Therapy

Targeting protein lactylation in cancer therapy holds promising potential. Lactylation is a key hallmark of metabolic reprogramming in tumor cells, especially under hypoxic conditions, where tumor cells produce large amounts of lactate through glycolysis, resulting in protein lactylation [172]. This modification can alter protein function, stability, and interactions, thereby promoting tumor initiation and progression. Consequently, targeting protein lactylation may disrupt the metabolic advantages of cancer cells, potentially inhibiting tumor growth and metastasis [26]. However, this therapeutic strategy faces several challenges. First, the regulatory mechanisms of lactylation remain unclear, and lactylation patterns may vary across different cancer types, raising questions about the generalizability and specificity of targeting lactylation. Additionally, since lactate is a byproduct of normal metabolic processes, broadly inhibiting lactylation could have adverse effects on normal cells, leading to undesirable side effects. Moreover, there is currently a lack of specific inhibitors or agonists for lactylation, and developing efficient and safe targeted therapies remains a major challenge. The roles of lactylation appear to vary significantly among different cancer types, making it challenging to generalize findings and develop universally applicable therapies [8]. Due to the involvement of lactate in multiple biological pathways, targeting lactylation could lead to unintended off‐target effects, including disruptions in normal cellular metabolism and immune function. Thus, although targeting protein lactylation shows potential in cancer therapy, its clinical application will require further basic research and clinical trials to ensure its safety and efficacy.

## 8 Conclusion and Perspective

The discovery of protein lactylation has unveiled a novel type of PTM, characterized by the addition of lactyl groups to lysine residues, which participates in the regulation of various cellular physiological functions. The regulatory factors of lactylation are complex, involving changes in intracellular lactate levels and the catalytic actions of specific enzymes. The methods for identifying lactylation sites are continually evolving, enabling a more precise understanding of its distribution and function in different proteins. Under physiological conditions, lactylation plays crucial roles in gene expression regulation, cellular metabolism, inflammatory response, and immune regulation. However, abnormal lactylation under pathological conditions can lead to the development and progression of various diseases. For instance, in tumors, lactylation not only promotes the proliferation and migration of tumor cells but also participates in remodeling the tumor immunosuppressive microenvironment, enhancing tumor immune evasion. Additionally, lactylation plays a critical role in inflammation and fibrosis, central nervous system diseases, cardiovascular diseases, and pathogenic microbial infections.

With the ongoing advancements in histone research, protein lactylation modifications have increasingly garnered the attention of researchers. Both histone and nonhistone proteins can undergo lactylation, which can occur through L‐lactylation or D‐lactylation pathways—both of which are prevalent in biological systems [12]. Lactylation modifications play significant roles not only in normal physiological activities but also in various human diseases, either directly or indirectly. Lactate production and metabolism influence the lactylation process, and gene expression, neuronal excitation, and cross‐talk can also affect lactylation modifications. Lactate, as an intermediate product of glycolysis, is a crucial regulatory factor involved in tumor immune microenvironments, tumor resistance, inflammatory diseases, and immune homeostasis regulation [93]. The discovery of lactate as a substrate for lactylation modifications and the study of its functions and regulatory mechanisms in various disease processes offer new insights into the impact of lactate on human physiological and pathological processes. As a novel PTM, understanding protein lactylation's roles and mechanisms in physiological and pathological contexts is essential and can deepen our understanding of many diseases, including cancer [8]. Despite current research elucidating the roles of lactylation in cancer, AD, inflammation, fibrosis, stem cell maintenance, embryonic development, and neural regulation, the field of protein lactylation is still in its infancy. Identifying lactylation sites across multiple systems (especially human and mouse cells) and forming a comprehensive multispecies lactylation atlas could significantly advance research in protein lactylation and enhance our understanding of its functions. The discovery of protein lactylation introduces new biological and functional considerations regarding lactate's role. Drug development based on lactate metabolism and protein lactylation modifications offers new hope for treating various diseases, including cancer. As an important metabolic product of cellular respiration, lactate serves both as an energy source and a signaling molecule that regulates neuronal function, immune inflammation, and TMEs. Research indicates that lactate can induce lactylation modifications and promote increased levels of these modifications [68]. The accumulating evidence suggests that lactylation, including both histone and nonhistone lactylation, plays a significant role under both physiological and pathological conditions. Compared to the more commonly studied acetylation modifications, protein lactylation is relatively novel and requires further, extensive exploration. This includes identifying lactylation enzymes and delactylation enzymes, examining other nonhistone modifications, and understanding how nonhistone lactylation regulates biological activities. It is certain that lactylation provides new clues for understanding the mechanisms of diseases such as cardiovascular disease, cancer, inflammation, and pulmonary fibrosis. For cardiovascular diseases, in addition to myocardial infarction, conditions such as myocardial hypertrophy and heart failure can lead to metabolic reprogramming of myocardial cells following ischemia and hypoxia [164]. Whether lactylation levels in myocardial cells increase and affect disease progression is an important area of research. Protein lactylation not only opens a new domain for PTM research but also offers new directions for studies in cancer and immunology. Additionally, the discovery of histone lactylation expands our understanding of the interplay between metabolic processes and epigenetic modifications. However, the specific mechanisms of lactate uptake and utilization in cancer cells, and the interactions between protein lactylation, metabolic reprogramming, and immune suppression, remain unclear. Further elucidation of these mechanisms is crucial and could drive the development of new anticancer therapies. Research into protein lactylation in neurological diseases is still in its early stages, and the specific molecular mechanisms linking lactylation with these diseases need further validation and exploration. We eagerly anticipate more research and discoveries to reveal the detailed principles of lactylation modifications, which could provide new approaches and methods for treating neurological diseases and offer more effective therapeutic targets.

Research on the regulatory effects of lactylation in cancer is predominantly focused on histone lactylation, with less emphasis on nonhistone lactylation, which may become a future research hotspot. While most studies suggest that lactylation promotes tumor development, some research indicates that lactylation may inhibit tumors, highlighting the complex and diverse regulatory effects of lactylation on cancer [64].

Future work could address the following areas to further elucidate the functional mechanisms of lactylation in both physiological and pathological conditions. First, expanding research on metabolic diseases, such as diabetes and obesity, could provide insights into how lactylation regulates key metabolic pathways and cellular energy homeostasis. Second, investigating lactylation in cardiovascular diseases, including atherosclerosis and heart failure, may reveal its role in vascular inflammation, lipid metabolism, and myocardial remodeling. Third, studying lactylation in cancer could deepen our understanding of its involvement in tumor progression, immune evasion, and therapeutic resistance, potentially identifying novel biomarkers or therapeutic targets. Additionally, exploring lactylation in neurodegenerative diseases and inflammatory disorders may uncover its contributions to neuroinflammation, neuronal function, and immune responses. Advancing detection techniques and developing specific modulators of lactylation will be critical for translating these findings into clinical applications. Future research should focus on the regulatory mechanisms of lactylation modifications to systematically understand their roles in malignant tumors and other diseases, providing a theoretical foundation for developing novel targeted cancer therapies.

Finally, future work should explore the role of lactylation in nonhistone proteins and its impact on less common diseases. While histone lactylation has been extensively studied in gene regulation, lactylation of nonhistone proteins may play crucial roles in cellular signaling, metabolic regulation, and protein stability. Investigating how lactylation affects key transcription factors, enzymes, and structural proteins could provide deeper insights into its functional diversity. Additionally, research on lactylation in less common diseases, such as rare metabolic disorders, autoimmune diseases, and neurodevelopmental conditions, may uncover novel mechanisms linking lactate metabolism to disease pathogenesis. Developing advanced detection techniques and functional studies will be essential for understanding these emerging aspects of lactylation and their potential clinical relevance.

In summary, targeting lactate metabolism and lactylation‐related enzymes for cancer therapy holds potential applications. By inhibiting key enzymes involved in lactylation or regulating lactate metabolism, it is possible to intervene in the metabolism and function of tumor cells, disrupt the TME, and thereby improve therapeutic outcomes. This provides new insights for the development of novel combinatorial targeted and immunotherapeutic strategies, which are expected to play a significant role in future cancer treatments. Further research into the mechanisms and functions of protein lactylation will help us better understand disease pathogenesis and provide a theoretical basis and practical guidance for clinical therapy.

## Author Contributions

Xi Chen, Yixiao Yuan, and Fan Zhou wrote the main manuscript text and prepared Figures. Lihua Li, Jun Pu, and Yong Zeng analyze the literature in depth, optimize the topic and structure. Lihua Li developed the conception of the topic. Xiulin Jiang, Jun Pu, and Yong Zeng supervised, reviewed, and revised the written manuscript. All authors reviewed the manuscript.

## Conflicts of Interest

The authors declare no conflicts of interest.

## Ethics Statement

The authors have nothing to report.

## Data Availability

The data that support the findings of this study are available in the article.
