# Correction to “Lactylation: from Homeostasis to Pathological Implications and Therapeutic Strategies”

**DOI:** 10.1002/mco2.70776

**Published:** 2026-05-29

X. Chen, Y. Yuan, F. Zhou, et al., “Lactylation: From Homeostasis to Pathological Implications and Therapeutic Strategies,” *MedComm* 6, no. 6 (2025): e70226, https://doi.org/10.1002/mco2.70226.

On Page 6, in the third paragraph, the statement ″This delayed temporal dynamic effect has been metaphorically described as a “lactate clock.” should have been supported by the following reference [1]. On Page 6, in the fourth paragraph, the statement “The level of lactylation can be influenced by various factors, including the production and transport of substrate lactate, the acyltransferases providing the lactyl group, neural excitation, gene expression, and the stimulation of certain traditional Chinese medicine components” should have been supported by the following reference [2]. On Page 6, in the fifth paragraph, the statement “Zhang et al. proposed that lactylation is derived from lactate; methods such as using gamma‐interferon combined with lipopolysaccharide or bacterial stimulation can increase lactate production in cells” should have been supported by the following reference [3]. On Page 7, in the second paragraph, the statement “However, Hagihara et al. induced histone H1 lactylation in mouse neuronal cells using different methods and then treated them with the MCT2 inhibitor α‐cyano‐4‐hydroxycinnamate (4‐CIN) and the more selective MCT1/2 inhibitor AR‐C155858, finding no significant changes in lactylation levels, suggesting the possible concurrent occurrence of an indirect lactylation pathway not inhibited by MCT inhibitors” should have been supported by the following reference [4]. On page 7 in the third paragraph, the statement “The acetyltransferase family has broad acyltransferase activity, utilizing different acyl‐CoAs as substrates for lysine acylation of various histones.” should have been supported by the following reference [5]. On Page 8, in the third paragraph, the statement “The study of lactylation's crosstalk with other modifications is still in its early stages.” should have been supported by the following reference [6]. On page 8 in the third paragraph, the statement “Evidence shows a close relationship between lactylation and acetylation.” should have been supported by the following reference [7]. On page 8 in the third paragraph, the statement “Histone lactylation and acetylation exhibit a high degree of spatial overlap, and lactylation levels increase as acetylation decreases” should have been supported by the following reference [1]. On Page 8, in the third paragraph, the statement “Lactylation serves as an intermediary linking metabolism and epigenetics, with crosstalk phenomena present in its modification process” should have been supported by the following reference [8].

The omission does not affect the overall conclusions of the paper. The authors apologize for this oversight.

Xiulin Jiang's current contact details: xiulinjiang17@163.com, UF Health Cancer Center, University of Florida, Gainesville, FL, USA.
